# Genomic and epigenomic *BRCA* alterations predict adaptive resistance and response to platinum-based therapy in patients with triple negative breast and ovarian carcinomas

**DOI:** 10.1126/scitranslmed.abn1926

**Published:** 2022-07-06

**Authors:** Francesca Menghi, Kalyan Banda, Pooja Kumar, Robert Straub, Lacey Dobrolecki, Isabel V. Rodriguez, Susan E. Yost, Harshpreet Chandok, Marc R. Radke, George Somlo, Yuan Yuan, Michael T. Lewis, Elizabeth M. Swisher, Edison T. Liu

**Affiliations:** 1 The Jackson Laboratory for Genomic Medicine; Farmington, CT 06030, USA; 2 Division of medical oncology, UW Medical Center; Seattle, WA, 98195, USA; 3 Clinical research division, Fred Hutchinson Cancer Research Center; Seattle, WA 98109, USA; 4 The Lester and Sue Smith Breast Center; Houston, TX, 77030, USA.; 5 Department of Obstetrics and Gynecology, UW Medical Center; Seattle, WA, 98195, USA; 6 Division of Medical Oncology and Therapeutic Research, City of Hope Comprehensive Cancer Center; Duarte, CA 91010; 7 Departments of Molecular and Cellular Biology and Radiology, The Lester and Sue Smith Breast Center, Dan L Duncan Comprehensive Cancer Center; Houston, TX, 77030, USA.

## Abstract

Triple negative breast (TNBC) and ovarian carcinomas (OvCas) with *BRCA1* promoter methylation (*BRCA1*meth) respond more poorly to alkylating agents compared to those bearing mutations in *BRCA1* and *BRCA2* (*BRCA*mut). This is a conundrum given the biologically equivalent homologous recombination deficiency induced by these genetic and epigenetic *BRCA* perturbations. We dissected this problem through detailed genomic analyses of TNBC and OvCa cohorts and experimentation with patient-derived xenografts (PDXs) and genetically engineered cell lines. We found that despite identical downstream genomic mutational signatures associated with *BRCA1*meth and *BRCA*mut states, *BRCA1*meth uniformly associates with poor outcomes. Exposure of *BRCA1*meth TNBCs to platinum chemotherapy, either as clinical treatment of a patient, or as experimental in vivo exposure of preclinical PDXs, resulted in allelic loss of *BRCA1* methylation, increased *BRCA1* expression and platinum resistance. These data suggest that unlike *BRCA*mut cancers, where *BRCA* loss is a genetically ‘fixed’ deficiency state, *BRCA1*meth cancers are highly adaptive to genotoxin exposure and, through reversal of promoter methylation, recover *BRCA1* expression and become resistant to therapy. We further found a specific augmented immune transcriptional signal associated with enhanced response to platinum chemotherapy but only in patients with BRCA proficient cancers. We showed how integrating both the strength of this cancer immune signature and the presence of *BRCA* mutations results in more accurate predictions of patient response when compared to either HRD status or *BRCA* status alone. This underscores the importance of defining *BRCA* heterogeneity in optimizing the predictive precision of assigning response probabilities in TNBC and OvCa.

## One Sentence Summary

Dynamics of *BRCA1* promoter demethylation after genotoxic exposure induce chemoresistance in breast and ovarian cancers and adaptively alter therapeutic outcomes

## INTRODUCTION

We and others have previously described genome-wide mutational patterns resulting from homologous recombination deficiency (HRD), and specifically from loss of BRCA1 or BRCA2 (hereby referred to as BRCA) activity, (1–6). As loss of BRCA activity has been associated with high sensitivity to DNA inter-strand crosslinking agents, such as platinum-based chemotherapies, several studies have tried to assess the value of different forms of HRD-linked genomic alterations as predictors of treatment response in both triple negative breast (TNBC) and ovarian cancer (OvCa), with conflicting outcomes (7–13). These inconsistencies may be the result of different methods to clinically detect BRCA deficiency, different chemotherapeutic regimens, and different disease states such as primary or metastatic. However, there is growing suspicion that distinct biological processes may also be at play, especially in cancers where *BRCA1* deficiency is due to gene silencing through promoter methylation (14–16).

Herein, we sought to use precise functional measures of BRCA deficiency to reexamine genomic clinical data across primary TNBC and OvCa clinical cohorts, whose treatment regimen comprising a combination of platinum- and taxane-based chemotherapy provided a consistent basis for comparison. We specifically sought clinical cohorts of patients with TNBC and OvCa treated using the same chemotherapeutic combinations and with accessible genomic data so that genomic assessments of HRD, including the SBS3 mutational signature (1, 6), copy number-based HRD scores (11) and the Tandem Duplicator Phenotype (TDP) type 1 (4, 5), could be performed. Since TDP type 1 is currently the most precise measure for the specific form of HRD that results from *BRCA1* deficiency, the assessment of TDP provides a unique genomic forensic tool to ascertain critical exposure to BRCA1-specific deficiency in the development of TNBC and OvCa. We then used well characterized cohorts of PDX models and engineered cell line models of TNBC to further explore our clinical observations and to uncover more mechanistic insights. Our results show that tumors with pathogenic mutations in either *BRCA1* or *BRCA2* (*BRCA*mut), those with *BRCA1* silencing through promoter methylation (*BRCA1*meth), and those that are proficient for both *BRCA1* and *BRCA2* (wild type for both *BRCA1* and *BRCA2* and without *BRCA1* promoter methylation, hereby called non*BRCA*) are distinct clinical-therapeutic entities in their response to platinum chemotherapies. The mechanistic heterogeneity of response to DNA damaging chemotherapy across the different *BRCA* states can explain many of the inconsistencies seen in clinical trials where response outcomes are measured against genomic-based HRD assessments, and provides an approach to formulate more precise predictive markers for therapeutic response in TNBC and OvCa.

## RESULTS

### *BRCA1* mutation and methylation result in the same genomic mutational signatures but associate with different therapeutic response in TNBC

**Fig. 1.**
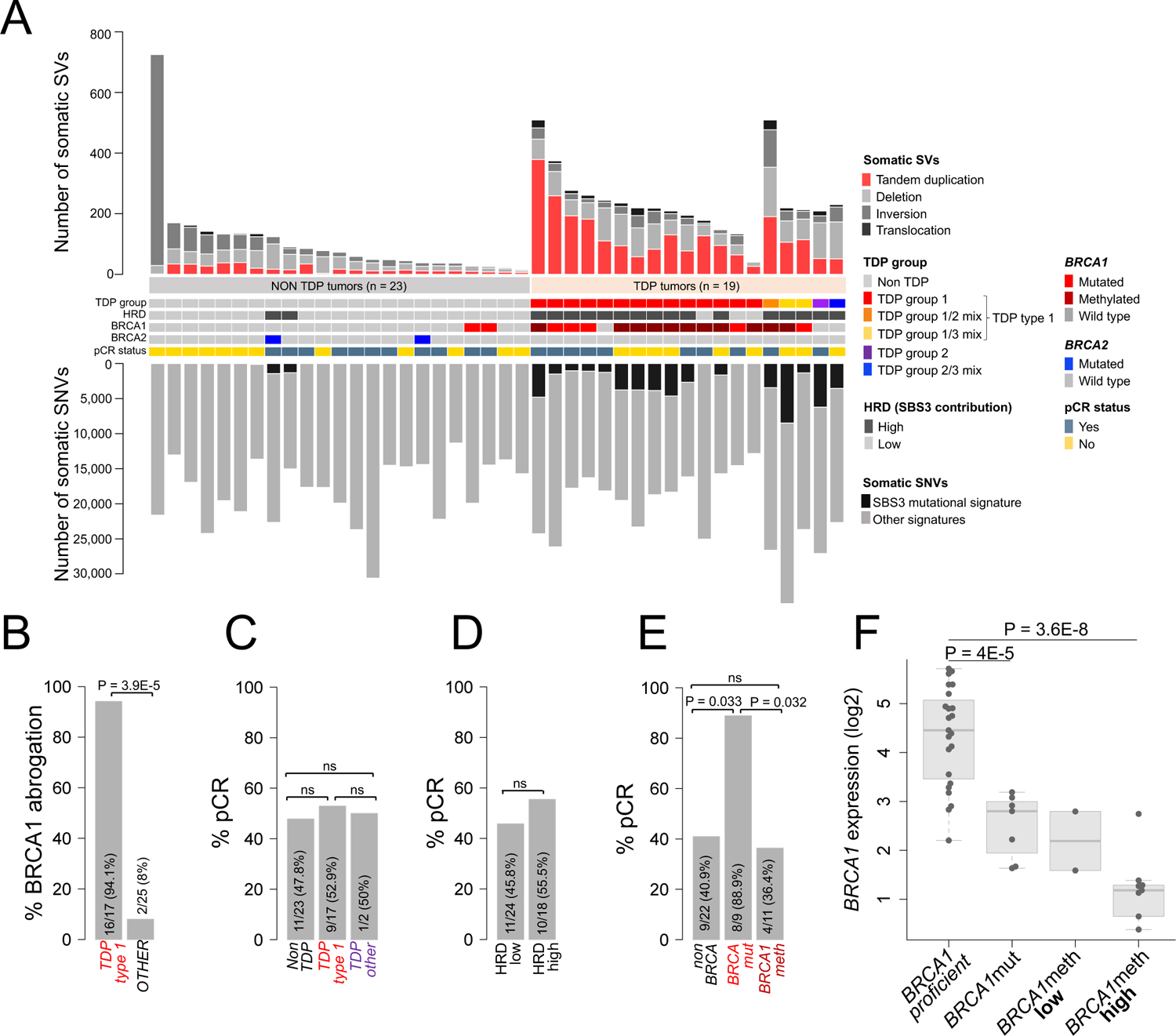
Genomic features and therapeutic response in the COH TNBC cohort. **(A)** Overview of the TNBC cohort genomic and clinical features. Samples are sorted based on their TDP group assignment. **(B)** Association between BRCA1 abrogation (by mutation or promoter methylation) and TDP status (TDP type 1 comprises all TDP subgroups with short span tandem duplications; TDP groups 1, 1/2 mix and 1/3 mix); the *P* value was determined by logistic regression. **(C-E)** Associations between TDP status (**C**, ‘TDP other’ refers to any TDP group other than TDP type 1), HRD status (**D**) and *BRCA* mutation status (**E**) with pCR rates; the *P* value was determined by logistic regression. **(F)** *BRCA1* gene expression across the COH TNBC cohort as a function of *BRCA1* status. *BRCA1* methylation was assessed using MS-ddPCR, with low and high methylation defined based on a 60% methylation threshold. The *P* value was computed by Student’s t-test (two-tailed).

To test the role of *BRCA* status and its genomic mutational signatures in the prediction of optimal response to platinum-based therapy, we assessed the genomic characteristics of a cohort of 42 patients with early stage TNBC, from the City Of Hope National Medical Center (COH TNBC cohort (17), Fig. 1A and tables S1–2). This cohort was selected because it was a phase II neoadjuvant trial where all patients received a total of four cycles of the carboplatin/NAB-paclitaxel doublet treatment and pre-treatment cancer specimens were available for whole genome and transcriptome analyses. We first assessed TDP type 1 status, which is a collective term that comprises all three BRCA1-linked TDP variations, including TDP group 1, TDP group 1/2 mix and TDP group 1/3 mix, as a genomic forensic tool to ascertain critical exposure to BRCA1-specific deficiency during tumor development. Consistent with our previous observations (4), TDP type 1 cancers were strongly associated with *BRCA1* mutation or promoter methylation as determined by methylation-specific PCR (MSP), with BRCA1 deficiency observed in 94.1% (16/17) of TDP type 1 cancers but only in 8% (2/25) of non TDP type 1 cancers (P = 3.9E-5, Fig. 1B). However, there was no association between TDP type 1 status and pathological complete response (pCR, Fig. 1C and table S3), which is commonly considered as the measure of optimal response in TNBC cohorts, and which leads to long term survival (18). Similarly, we found no significant association between HRD status, measured as a high proportion of the SBS3 mutational signature, and pCR (Fig. 1D and table S3). Breast cancers from both *BRCA1* and *BRCA2* germline mutation carriers have been previously shown to be sensitive to platinum-based chemotherapeutic regimens (19, 20). Since, we found that all patients with *BRCA2* mutant cancers in the COH cohort achieved pCR (table S2), and all our subsequent analyses also showed improved outcomes in *BRCA2* mutant cancers (table S3), we grouped TNBCs harboring *BRCA1* and *BRCA2* pathogenic mutations together as *BRCA*mut cancers. Despite the modest sample size, we observed signficantly higher pCR rates in patients with *BRCA*mut cancers, but not *BRCA1*meth cancers, than in those with either *BRCA1* and *BRCA2* proficient cancers (*P* = 0.033, Fig. 1E). By contrast, the pCR rate was low in patients with *BRCA1*meth cancers (Fig. 1E) and it was comparable with that of patients with non*BRCA* cancers. *BRCA1*mut and *BRCA1*meth TNBCs shared comparable average numbers of tandem duplications and shared similar span size distribution profiles (figs. S1A and S1B) implying equivalent loss of BRCA1 activity across the two modes of functional abrogation.

Our initial assessment of *BRCA1* promoter methylation used the MSP approach which is not quantitative. This raises the possibility that the poor performance of the individuals with *BRCA1*meth cancers may be due to incomplete methylation. To resolve this, we employed methylation-specific droplet digital PCR (MS-ddPCR) to reassess the *BRCA1* methylation status of the *BRCA1*meth cancer genomes in a quantitative manner (21). Nine of the 11 *BRCA1*meth cases were considered highly (or fully) methylated based on methylation assessments previously proposed for ovarian cancers, whereas two cancers scored below the threshold for high methylation (21). Although the highly methylated cancers showed an average of more than an 8-fold decrease in *BRCA1* gene expression compared to *BRCA1* proficient cancers (*P* = 3.6E-8, Fig. 1F), patients with these methylated *BRCA1* cancers did poorly with only 3/9 achieving pCR (table S2).

### *BRCA* abrogation and specific response to platinum across PDX models of human TNBC

**Fig. 2.**
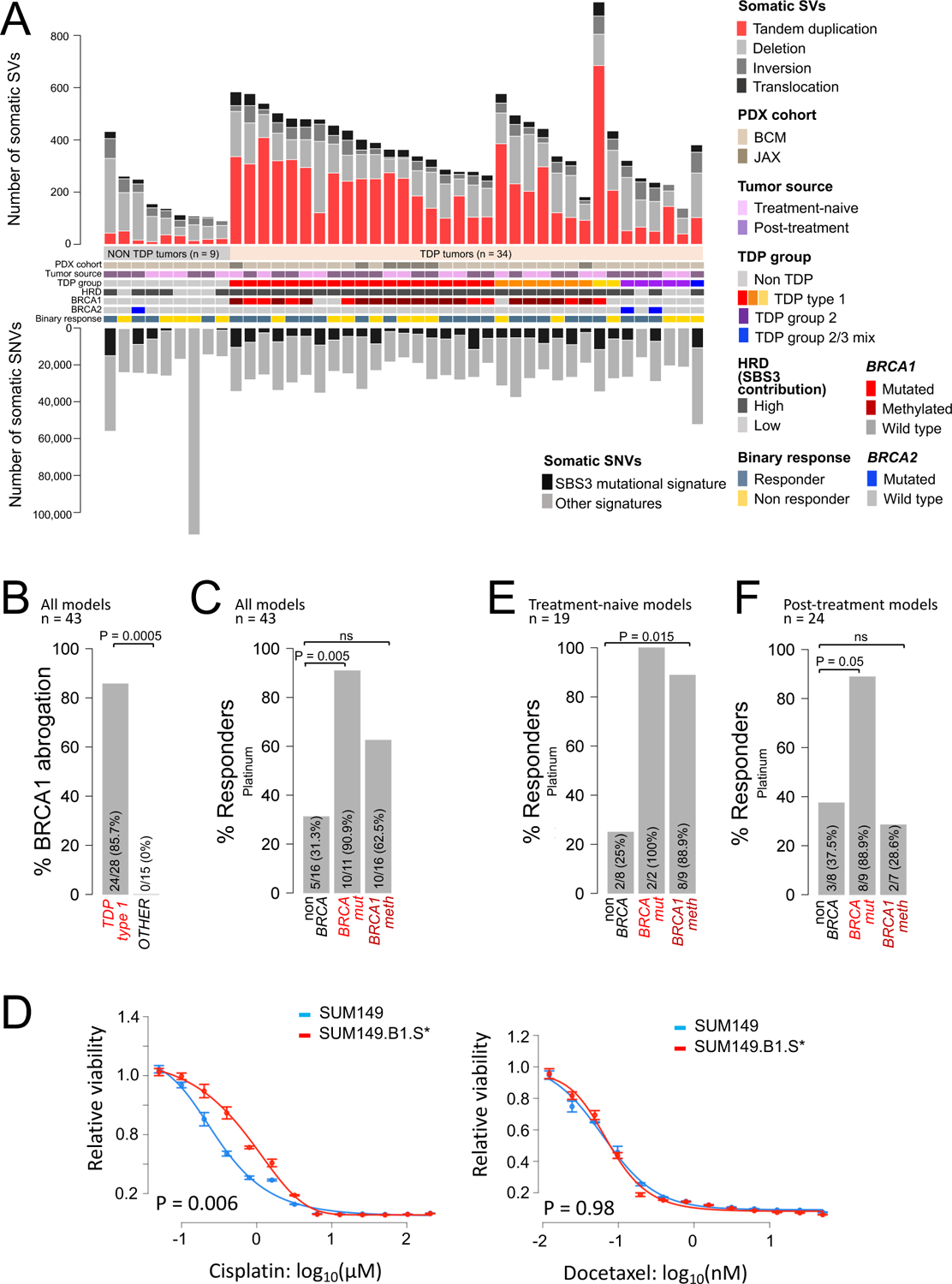
Genomic features and chemotherapy response in TNBC PDXs and cell lines. **(A)** Overview of genomic features and chemotherapy response in the TNBC PDX cohort. Samples are sorted based on their TDP group assignment. **(B)** Association between BRCA1 abrogation and TDP status; P value was determined by Fisher’s exact test. **(C)** Percentage of platinum responders as a function of *BRCA* status relative to the entire PDX cohort; the *P* value was determined by Fisher’s exact test. (**D)** Cisplatin and docetaxel IC_50_ curves relative to the SUM149/SUM149.B1.S* isogenic cell lines. One representative example of four biological replicate experiments is shown in each graph (IC_50_ values relative to all four biological replicates are reported in table S5). Data are presented as mean values and standard errors of the three technical replicates. Significance values were calculated using the Student’s t-test (two-tailed) to compare IC_50_ values from the four biological replicates across the two cell lines. (**E-F**) Percentage of platinum responders as a function of *BRCA* status for the subsets of PDXs established from ether treatment-naïve (**E**) or post-treatment patient tumors (**F**). The *P* value was determined by Fisher’s exact test.

One of the objectives of our study was to ask the key determinants of platinum sensitivity. However, most clinical studies, including those investigated herein, use combination chemotherapy which makes it difficult to parse out the effects of individual agents. Moreover, relevant clinical studies examine the clinical endpoint of pCR after four cycles of neoadjuvant chemotherapy, whereas using patient-derived xenograft (PDX) models, we can resolve the interim sensitivity dynamics of consistently sized tumors after a short course of chemotherapy. This allows for greater resolution to the question of relative sensitivity to any experimental agent. To this end, we analyzed a cohort of 43 TNBC PDXs with known response to both single agent platinum (either carboplatin, n = 36, or cisplatin, n = 7) and single agent docetaxel. This PDX cohort comprised 11 *BRCA*mut PDXs carrying deleterious mutations in either *BRCA1* (n = 8) or *BRCA2* (n = 3), 16 *BRCA1*meth PDXs and 16 non*BRCA* PDXs (Fig. 2A, tables S1–2). As expected, we confirmed the association between *BRCA1* status and TDP type 1, with BRCA1 abrogation affecting 24/28 TDP type 1 PDX models but none of the 15 non TDP type 1 models (*P* = 0.0005, Fig. 2B). After one cycle of platinum treatment, we compared in vivo tumor growth rates across the different *BRCA* groups. We found that only 31.3% (5/16) of non*BRCA* PDX models showed therapeutic response, defined as a > 5% average decrease in tumor growth rate compared to the control arm. By contrast, 90.9% of *BRCA*mut PDXs (10/11, *P* = 0.005) had a response, including 7/8 *BRCA1*mut and 3/3 *BRCA2*mut models (Fig. 2C, table S3). *BRCA1*meth PDXs showed an intermediate response trend with 62.4% (10/16) of responders, although this was not statistically significant when compared to the non*BRCA* PDX models (Fig. 2C, table S3). Similar response profiles were obtained when response was assessed using modified RECIST criteria, where PDXs that demonstrated RECIST values equivalent to complete or partial response were designated as responders (table S4). Although both TDP type 1 and HRD-high TNBC PDX models showed a trend towards better response to platinum compared to non TDP and HRD-low PDX models, respectively, these differences were also not statistically significant (fig. S2A and B, table S3). We observed no differences in response among the three *BRCA* classifications when the same PDXs were treated with docetaxel as single agent (table S4). These data suggested that *BRCA* status is associated with differential response to platinum agents and not to taxanes.

To further validate the specificity of the differential sensitivity to platinum in TNBC, we tested the chemotherapy responsiveness of the SUM149 human TNBC cell line that harbors pathogenic mutations in both *BRCA1* and *TP53* as compared to its derivative SUM149.B1.S* cell line where BRCA1 activity was restored by introducing a secondary mutation using CRISPR-Cas9-mutagenesis, which reinstates the correct open reading frame (22). The SUM149 parental cell line was 2.6 times more sensitive to cisplatin compared to the SUM149.B1.S* derivative (*P* = 0.006, Fig. 2D, table S5). By contrast, the two cell lines did not differ in their IC_50_ values when treated with the taxane docetaxel (Fig. 2D, table S5). These experiments in the PDX and the engineered cell lines support the notion that *BRCA1* deficiency is the cause of differential sensitivity to platinum-based agents.

We then explored whether the clinical origin of the PDX models would affect response behavior. Our PDX cohort comprises both treatment-naïve models established from patient cancers prior to any treatment (n = 19), and post-treatment models established from patient cancers that have been clinically exposed to chemotherapy (n = 24). Treatment-naïve *BRCA1*meth PDX models had significant better response rates compared to their non*BRCA* counterparts, with 88.9% (8/9) and 25% (2/8) response rates in the *BRCA1*meth and non*BRCA* treatment-naïve PDX subgroups, respectively (*P* = 0.015, Fig. 2E). However, there was no significant difference in response rate between the post-treatment *BRCA1*meth PDXs and the non*BRCA* post-treatment PDX subgroup, with 28.6% (2/7) and 37.5% (3/8) of responders in the *BRCA1*meth and non*BRCA* PDX subgroups, respectively (Fig. 2F). On the other hand, *BRCA*mut PDXs were uniformly good responders, with 2/2 responders in the treatment-naïve PDX group and 8/9 responders in the post-treatment PDX group (Figs. 2E and F). Importantly, no significant differences in response rates were observed when the PDXs were treated with docetaxel (table S4). These results indicate that the ability of *BRCA1*meth TNBC PDXs to respond to platinum therapy is strictly associated with their treatment-naïve status. However, the poor response for non*BRCA* and the good response in *BRCA*mut PDXs were sustained regardless of their original treatment status. This suggests that *BRCA1*meth tumors can more readily change their sensitivity profiles after exposure to chemotherapy whereas *BRCA1*mut and non*BRCA* tumors have more stable response phenotypes.

### Loss of *BRCA1* promoter methylation and induction of *BRCA1* expression in TNBC PDXs after platinum-based chemotherapy

**Fig. 3.**
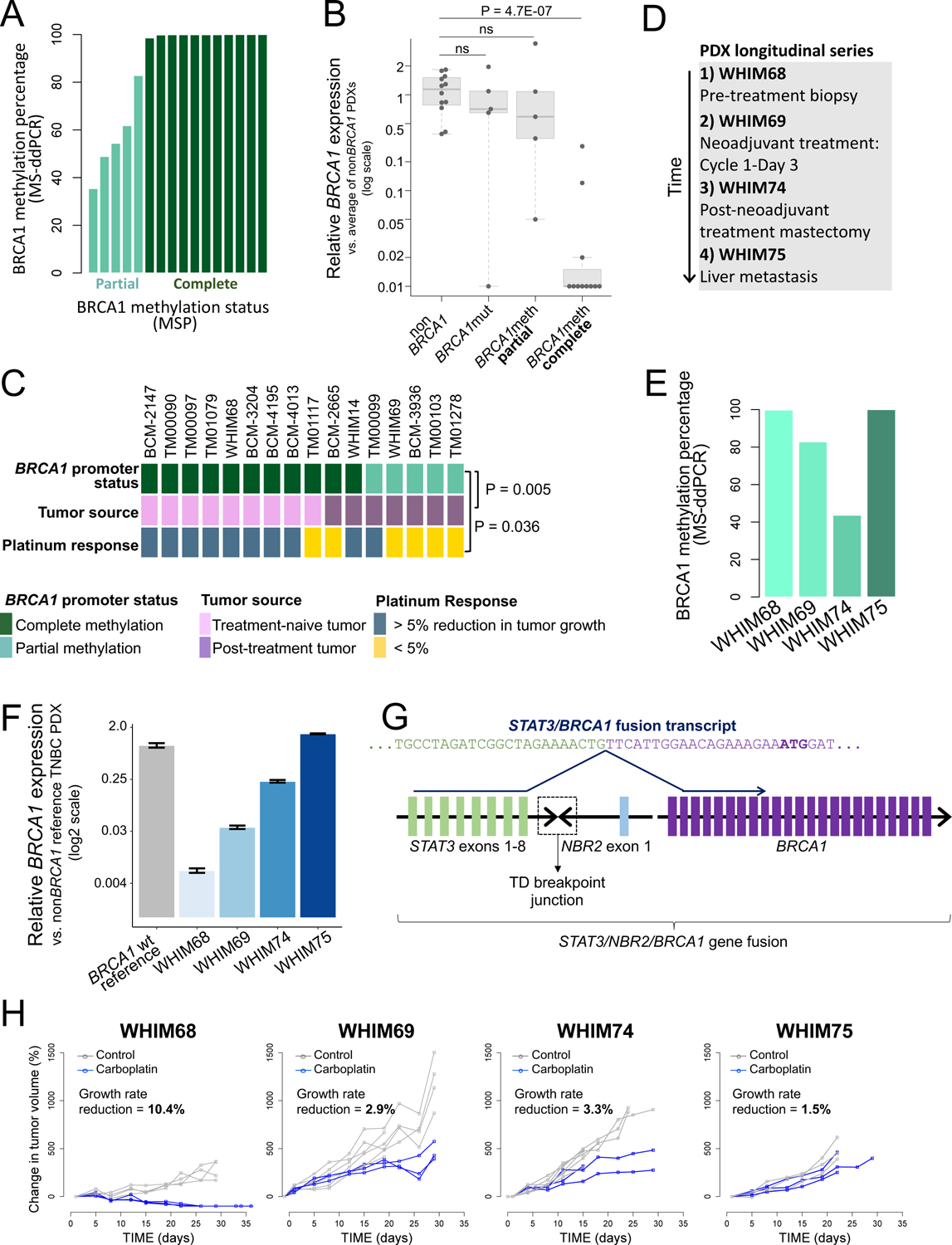
*BRCA1* methylation profiles and chemotherapy response in TNBC PDXs. **(A)** Percentage of *BRCA1* methylation for the 16 *BRCA1*meth TNBC PDX models computed by MS-ddPCR. Samples are sorted by increasing degree of methylation and color coded based on the partial vs. complete methylation status assigned by MSP. **(B)** *BRCA1* gene expression (qPCR) as a function of *BRCA1* status in TNBC PDX models. The average value of all the assessed non*BRCA1* PDX models was used as the calibrator in assessing expression fold changes. *P* values were computed by Student’s t-test (two-tailed). **(C)** *BRCA1* methylation status, tumor source and platinum-based therapy response for 16 *BRCA1*meth TNBC PDX models. The *P* value was determined by Fisher’s Exact test. **(D)** Tumor source for the four TNBC PDX models of the WHIM PDX longitudinal series. **(E)** Percentage of *BRCA1* methylation for the four PDX models of the WHIM PDX longitudinal series (MS-ddPCR). **(F)** *BRCA1* expression for the four PDX models of the WHIM PDX longitudinal series, assessed by qPCR. A non*BRCA1* TNBC PDX was used as the calibrator. Data are presented as mean values and standard errors of three technical replicates. **(G)** Schematic representation of the genomic fusion and corresponding fusion transcript involving the *STAT3*, *NBR2* and *BRCA1* genes in the WHIM75 PDX model. Colored blocks represent individual exons and are not drawn to scale. A dashed box indicates the location of the tandem duplication breakpoint junction between *STAT3* intron 8–9 and *NBR2* intron 1–2. In the *STAT3/BRCA1* fusion transcript, the last nucleotide of *STAT3* exon 8 is fused to the first nucleotide of *BRCA1* exon 2, which carries the BRCA1 translation initiation codon (ATG, highlighted in bold). **(H)** Tumor growth curves of the four TNBC PDX models of the longitudinal series. The average percentage reduction in tumor growth rate for the carboplatin arm compared to the control arm is reported on each graph. Responders are identified as PDX models showing a > 5% growth rate reduction.

MSP performed on the *BRCA1*meth PDX cohort revealed two modes of *BRCA1* promoter methylation: (a) complete methylation where there was no signal for the unmethylated PCR product and (b) partial methylation where there were two signals corresponding to both the methylated and the unmethylated PCR products (fig. S3A). When reassessed using MS-ddPCR, all of the 11 PDX models with complete methylation as determined by MSP scored > 98% in methylation percentage, whereas the five PDX models with only partial methylation showed methylation percentages ranging from 35.4% to 82.8% (Fig. 3A and table S2). We then asked whether these different modes of promoter methylation had an impact on *BRCA1* gene expression. We found that complete promoter methylation associated with a 99% decrease in the median *BRCA1* expression when compared to that of non*BRCA1* TNBC PDXs (*P* = 4.7E-07, Fig. 3B), whereas in partially *BRCA1*meth PDXs, this was not significant, with a median reduction in *BRCA1* expression values of only 41% (Fig. 3B). We also observed a significant association between the methylation status of the *BRCA1*meth models and the PDX tumor source: the majority of models with complete methylation of the *BRCA1* promoter were treatment-naïve (n = 9/11, 81.8%), whereas all of those with partial methylation (5/5) had been established from post-treatment tumors (*P* = 0.005, Fig. 3C). Furthermore, *BRCA1*meth PDXs with complete methylation had significantly higher response rates compared to those with partial methylation (*P* = 0.036, Fig. 3C). When PDX response was assessed as a continuous metric of percent reduction in growth rate comparing the platinum arm to the control arm, there was a statistically significant association between the degree of promoter methylation and post treatment tumor growth-rate reduction (r = 0.53, *P* = 0.036, fig. S3B).

We further investigated the relationship between *BRCA1* methylation status, treatment exposure and platinum sensitivity, using four distinct TNBC PDX models, WHIM68, WHIM69, WHIM74 and WHIM75, established from sequential breast cancer biopsies and surgical specimens of the same patient donor, hereby referred to as the WHIM PDX longitudinal series (Fig. 3D). WHIM68 was established from a pre-neoadjuvant treatment breast biopsy and is treatment-naïve, WHIM69 was derived from a research biopsy taken at day 3 of the first cycle of neoadjuvant treatment with carboplatin and docetaxel; WHIM74 was derived from a mastectomy specimen obtained after the completion of the neoadjuvant course; and WHIM75 was established from a liver metastasis. This PDX series allowed us to ask whether chemotherapeutic treatment of an individual patient with TNBC in the clinical setting would result in the progressive loss of *BRCA1* promoter methylation and the augmentation of steady state *BRCA1* expression. MS-ddPCR analysis showed that the amount of *BRCA1* methylation progressively reduced from ~100% in WHIM68, to 82.8% in WHIM69 and 43.7% in WHIM74 (Fig. 3E). *BRCA1* gene expression, as assessed by quantitative PCR, was nearly undetectable in the WHIM68 model, but progressively increased in each successive PDX model, reaching amounts comparable to the BRCA1 proficient state in WHIM75 (Fig. 3F). The results from the first three PDXs in the series suggest that loss of *BRCA1* methylation can occur shortly after a single exposure to chemotherapy in the patient, and that the resultant cancers progressively increase *BRCA1* expression after successive chemotherapeutic exposures. By contrast, WHIM75, derived from a liver metastasis, appeared to be an outlier, in that it showed normal *BRCA1* expression but retained a complete methylation profile (Fig. 3E–F). We hypothesized that the elevated expression of *BRCA1* in WHIM75 may be caused by a form of promoter bypass and scanned its genome for somatic rearrangements engaging the *BRCA1* locus. In a detailed genomic analysis, we found a ~800 Kb tandem duplication resulting in the fusion of the *NBR2* non-protein coding gene, which resides adjacent to the *BRCA1* promoter, with the *STAT3* gene, located several hundreds of Kbs upstream of *BRCA1* (fig. S4A). Using PCR and Sanger sequencing, we confirmed that this rearrangement is specific to WHIM75 and is not found in the other three PDX models from the same patient (fig. S4B). Sequence analysis of the breakpoint junction revealed a four nucleotide microhomology region, but no larger stretch of homology between the two gene fusion partners (fig. S4C). Based on the exonic structure of the genes involved in the rearrangement, we predicted that the duplication would result in the production of a fusion transcript between the *STAT3* and the *BRCA1* genes, by placing the *BRCA1* gene under the control of the *STAT3* promoter, effectively bypassing the negative regulation of the hypermethylated *BRCA1* promoter. Reverse transcription PCR and Sanger sequencing confirmed the presence of a *STAT3/BRCA1* fusion transcript, where the last nucleotide of *STAT3* exon 8 is fused to the first nucleotide of *BRCA1* exon 2, which carries the *BRCA1* translation initiation codon (Fig. 3G), and that its expression is specific to the WHIM75 PDX model (fig. S4D). A similar fusion has been previously reported when a TNBC *BRCA1*meth PDX was rendered resistant to cisplatin therapy in an experimental setting (23), but, to our knowledge, this is the first time that a similar mechanism of BRCA1 re-activation has been identified in a clinically-derived sample. The progressive re-expression of *BRCA1* in the WHIM PDX series, either by loss of methylation, such as in WHIM68 and WHIM74, or by promoter hijacking in WHIM75 was associated with resistance to carboplatin in vivo (Fig. 3H). The WHIM PDX series therefore showed that, upon chemotherapy exposure, re-activation of BRCA1 occurs in the clinical setting and is associated with acquired resistance to platinum-based therapy. That BRCA1 re-activation can be driven by alternative mechanisms in different *BRCA1*meth cancer tissues from a single patient, namely loss of *BRCA1* methylation at the primary cancer site and a genomic rearrangement placing the *BRCA1* gene under the control of a heterologous promoter in the metastasis to the liver, suggest that re-expression of *BRCA1* is a significant driver for chemotherapeutic insensitivity.

**Fig. 4.**
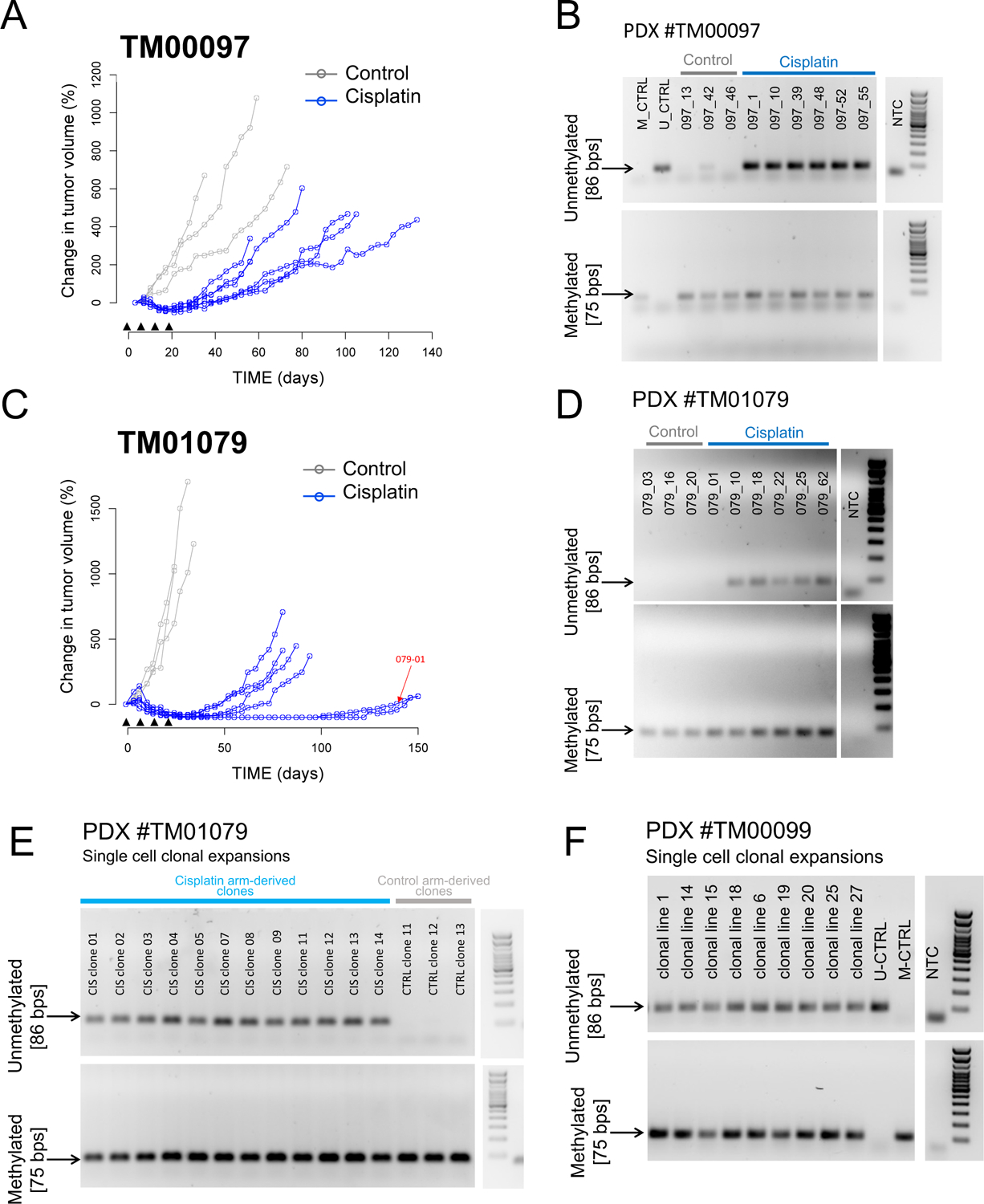
Assessment of *BRCA1* methylation and expression in TNBC PDXs treated with cisplatin in vivo. **(A)** Tumor growth curves for the TNBC PDX model TM00097, established from a treatment-naive patient tumor. Black arrow heads indicate the timing of the four weekly doses of cisplatin. **(B)** MSP results for vehicle tumors and cisplatin-treated tumor recurrences relative to PDX #TM00097. **(C)** Tumor growth curves for the TNBC PDX model TM01097, established from a treatment-naive patient tumor. Black arrow heads indicate weekly doses of cisplatin. A red arrow indicates the growth curve for animal #097–01, whose recurrent tumor maintained a full methylation profile, as assessed in (**D**). **(D)** MSP results for vehicle tumors and cisplatin-treated tumor recurrences relative to PDX #TM01079. **(E)** MSP results for fifteen single cell derived clonal expansions of the primary cultures established from tumor residuals from the cisplatin (*n* = 12) and control (*n* = 3) arm of the response study using the *BRCA1* fully methylated TNBC PDX #TM01079. **(F)** MSP results for nine single cell derived clonal expansions of the primary cultures established from an untreated tumor fragment of the *BRCA1* partially methylated TNBC PDX #TM00099. Although not exposed to chemotherapy as a PDX, #TM00099 was established from a post-treatment patient tumor.

Based on the *BRCA1* methylation patterns observed in the WHIM PDX series and in the PDX models derived from treatment-naïve or post-treatment patient cancers, we hypothesized that fully methylated *BRCA1*meth tumors would undergo demethylation after even short exposure to chemotherapy, resulting in restoration of *BRCA1* expression and resistance to platinum. To test this possibility, we selected a single completely methylated TNBC PDX model established from a treatment-naïve patient cancer (#TM00097) and subjected it to treatment in vivo with four weekly doses of either cisplatin or vehicle control, followed by regular monitoring of tumor recurrence (Fig. 4A). We found that only one of the three tumors in the control/untreated arm showed a weak unmethylated signal, whereas all six cisplatin recurrences showed the emergence of a strong unmethylated signal, clearly suggesting that even brief chemotherapeutic exposure can cause the epigenetic shift at the *BRCA1* promoter locus (Fig. 4B). Similar results were obtained with a second treatment-naïve and completely methylated PDX model (#TM01079), with all three tumors in the control arm maintaining complete methylation, but five out of six cisplatin recurrences showing the emergence of the unmethylated signal (Figs. 4C and D). Loss of methylation in both PDX models was accompanied by re-expression of the *BRCA1* gene to amounts often comparable to those found in non*BRCA* and *BRCA1*mut tumors (fig. S5A).

We next asked the question of whether compounds commonly used in the clinical treatment of patients with TNBC, such as doxorubicin, cyclophosphamide and docetaxel, can similarly cause the conversion from complete to partial *BRCA1* methylation and, consequently, restoration of *BRCA1* expression. To this end, we treated the #TM00097 TNBC PDX model with a combination of doxorubicin and cyclophosphamide, administered weekly for three weeks, followed by three weekly doses of docetaxel (AC→T). The AC→T regimen, mimicking a commonly used TNBC clinical protocol, was efficient in reducing tumor volume (fig. S6A). However, after ~60 days of drug holiday, tumors eventually relapsed. When tested for *BRCA1* promoter methylation, all three tumor residuals from the control arm maintained the complete *BRCA1* methylation profile, whereas all three tumor relapses from the AC→T arm converted to partial methylation (fig. S6B), similar to their cisplatin treated counterparts in the previous experiments. Again, loss of methylation in the AC→T relapses was associated with a significant increase in *BRCA1* expression (*P* = 0.002, fig. S6C). This demonstrates that standard non-platinum regimens commonly used in TNBC can also induce the partial methylation status associated with higher *BRCA1* expression and platinum insensitivity.

### Loss of *BRCA1* promoter methylation is due to demethylation of one *BRCA1* promoter allele

One proposed hypothesis for the loss of *BRCA1* methylation following chemotherapeutic treatment and the emergence of resistant tumor recurrences is the rapid expansion of a non-methylated subclone from a heterogeneous tumor cell population that contains *BRCA1*meth and non*BRCA* clones. To assess this possibility, we explored existing genomic data of tumor pairs where this question could be addressed. Patch et al. described the case of a patient (AOCS-091) initially diagnosed with a *BRCA1* methylated, platinum sensitive primary OvCa, who eventually developed a recurrent cancer that was unmethylated and chemo resistant (24). It was speculated that the recurrence may have originated from an independent original *BRCA1* proficient and promoter unmethylated, platinum resistant subclone, which was expanded during the chemotherapeutic treatment (24). Through a detailed genomic reanalysis of structural mutations in the AOCS-091 OvCa pair, we found that, despite the presence of private rearrangements, both the primary and the recurrent cancer genomes classified as TDP type 1 (fig. S4E). This indicated that the recurrent tumor originated from a subclone that, early in its evolutionary history, had experienced BRCA1 deficiency in order to generate BRCA1-related genomic mutational signatures. Given the absence of *BRCA1* gene mutations, we hypothesize that the resistant and unmethylated subclone was derived from the same genomic origin of its sensitive and *BRCA1* methylated primary counterpart, and that it eventually lost *BRCA1* methylation at a later timepoint.

We then took advantage of our *BRCA1*meth TNBC PDX models to test the hypothesis of active demethylation; namely conversion of one *BRCA1* promoter allele from the methylated to the unmethylated state following chemotherapeutic exposure. To this end, we established primary cell cultures from both an untreated fully *BRCA1* promoter methylated TM01079 PDX tumor and one of the partially methylated TM01079 relapses emerging from the single cycle of cisplatin treatment, as described above. Single cells were isolated from both primary cell cultures and expanded as clonal cell lines. MSP analysis of *BRCA1* methylation revealed that all of the clonal expansions from the untreated PDX primary culture (3/3) had complete methylation of the *BRCA1* promoter, confirming that a complete *BRCA1* methylation profile can be maintained in the absence of environmental exposures (Fig. 4E). On the other hand, all of the cisplatin-treated tumor derived clones (12/12) showed both methylated and unmethylated signals, suggesting heterozygous loss of *BRCA1* methylation at the cellular level (Fig. 4E). This loss of methylation after cisplatin treatment was accompanied by a significant increase in *BRCA1* expression (*P* = 0.0002, Fig. S5B). That all twelve clones isolated from the relapsed PDX tumor showed the same profile of heterozygous methylation suggests that demethylation of one *BRCA1* promoter allele occurred in all or a vast majority of the cells comprising the entire recurrent tumor. To confirm these clonal observations, we also established single cell clonal expansions from the TM00099 PDX model, a TDP type 1 TNBC established from a post-treatment patient tumor and exhibiting partial methylation at the *BRCA1* promoter when assessed in the bulk tumor (Fig. 3C). MSP analysis of each of the nine individual clonal lines established showed both methylated and unmethylated amplification products (Fig. 4F), again consistent with a pattern of heterozygous *BRCA1* methylation for each clone examined. These results suggest that the same allelic profile of epigenetic conversion at the *BRCA1* promoter experimentally induced in the TM01079 model by in vivo platinum exposure can also manifest in the clinical setting. Taken together, these results are consistent with the explanation that the loss of methylation in *BRCA1*meth tumors is the result of demethylation of one *BRCA1* promoter allele in most of the cells in the bulk tumor, and not through the expansion of a non-methylated subclone.

### *BRCA* gene mutations, and not *BRCA1* methylation, are also strongly predictive of platinum-based therapy response in independent OvCa cohorts

**Fig. 5.**
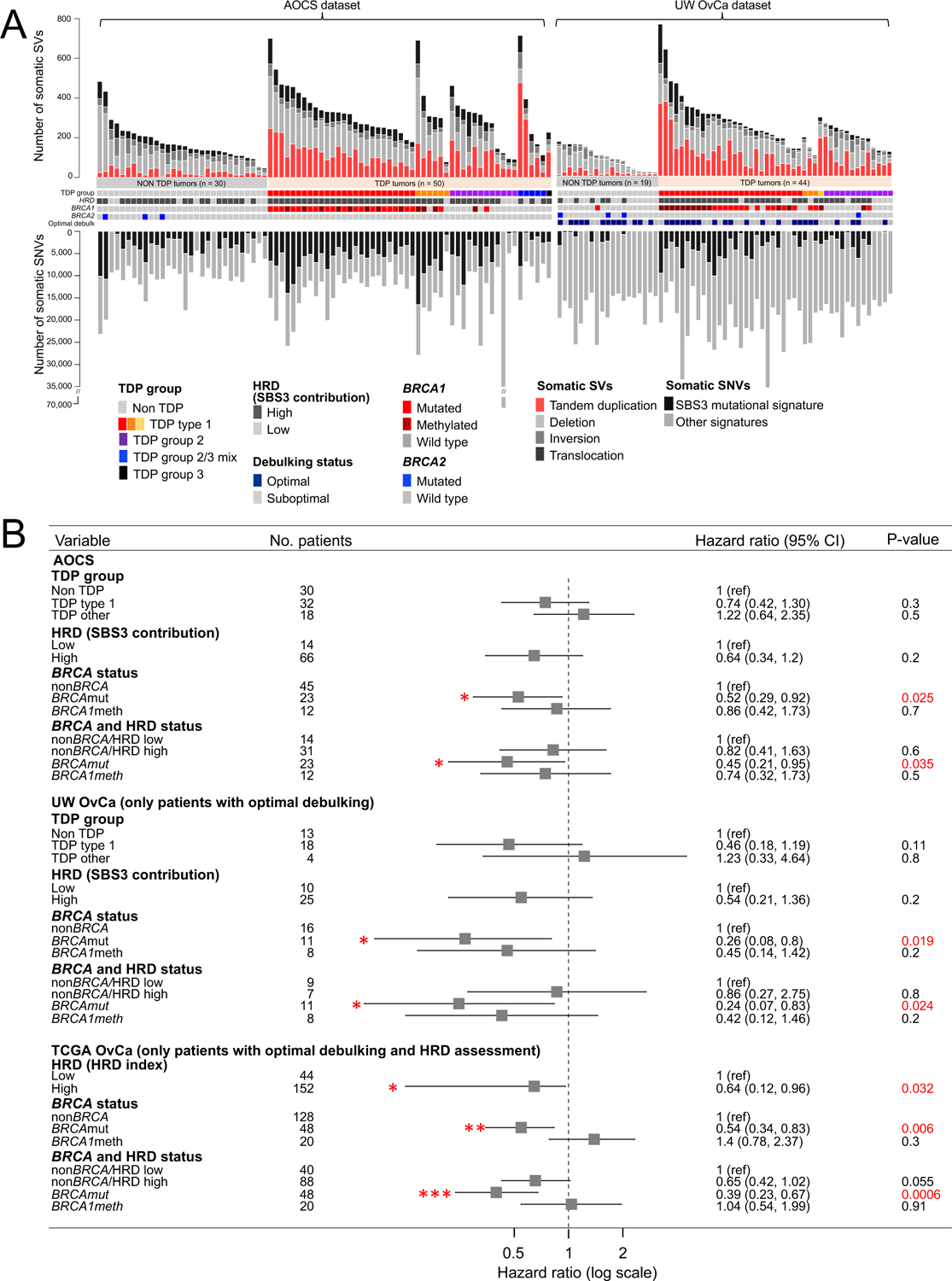
Genomic features and therapeutic response across independent cohorts of patients with OvCa. **(A)** Overview of the genomic and clinical features relative to the AOCS and the UW OvCa cohorts. Samples are sorted based on their TDP group assignment. **(B)** Forest plot for the AOCS, UW OvCa and TCGA OvCa cohorts, showing hazard ratios, their 95% confidence intervals, and *P* values based on a univariate Cox proportional hazards regression model testing whether overall survival associates with TDP status, HRD status, *BRCA* status or a combination of *BRCA* and HRD status. Significant *P* values are highlighted in red. * *P* < 0.05; ** *P* < 0.01; *** *P* < 0.001.

We previously determined that BRCA1 deficiency results in the same TDP type 1 configuration in both TNBC and OvCa pointing to similarities of biological effects of *BRCA* status in these two conditions (4). We therefore sought to recapitulate in OvCa cohorts our findings in TNBC that specific *BRCA* configurations are predictive of patient response to platinum-based chemotherapy. Despite their genomic similarities, the clinical features of the two cancer types are very different, especially with respect to tumor burden at diagnosis and response assessment. At presentation, OvCa has higher tumor burden than TNBC, and the determination of therapeutic response in OvCa is not primarily by quantitative assessment of tumor shrinkage, such as assessment of pCR in TNBC, but by duration of response and overall survival, a less direct measure of chemo-responsiveness. We kept these differences in mind as we examined publicly available datasets for OvCa cohorts with complete genome sequence information and response assessment. We first analyzed the Australian Ovarian Cancer Study (AOCS) cohort which comprises 80 primary OvCas (24), including 32 TDP type 1 cancers, 30 of which exhibit some form of BRCA1 abrogation (Fig. 5A and fig. S7A). When patients with OvCa were stratified based on their TDP or HRD status there was no significant association with survival (Fig. 5B). However, patients with *BRCA*mut cancers showed a significant improved overall survival compared to those with non*BRCA* cancers (Hazard Ratio (HR) = 0.52, *P* = 0.025; Fig. 5B), whereas, again, *BRCA1* promoter methylation showed no significant survival advantage. Virtually all *BRCA*mut and *BRCA1*meth tumors have high HRD scores, but non*BRCA* tumors can be segregated into HRD-low and HRD-high subgroups. When we analyzed overall survival specifically in the non*BRCA* group, we found that patients with non*BRCA* HRD-high cancers did not have significantly improved outcomes compared to those with non*BRCA* HDR-low cancers (HR = 0.64, Fig. 5B). This again suggests that HRD alone in the absence of *BRCA* mutation and methylation status is not a good predictor of response.

Given the consistent signal from the AOCS cohort, we pursued more in-depth analysis in an independent OvCa dataset. To this end, we analyzed a new cohort of 63 patients with primary OvCa from the University of Washington Medical Center (UW OvCa cohort, Fig. 5A, tables S1–2), with detailed response data to therapy with a combination of carboplatin and paclitaxel and access to the primary cancer samples for detailed genomic analyses. In this cohort, BRCA1 deficiency was again significantly associated with the TDP type 1 configuration (*P* = 2.4E-7, fig. S7B), with similar proportions of *BRCA1*mut and *BRCA1*meth OvCas defining the BRCA1 deficient group (table S2). When we examined the correlation of overall survival with *BRCA* status in the UW OvCa cohort, again, we found that patients with *BRCA*mut OvCas achieved better survival when compared to patients with non*BRCA* OvCas (HR = 0.48, P = 0.046, fig. S7C). In this cohort, patients with *BRCA1*meth OvCa did show a trend for better outcome, although it was not statistically significant (HR = 0.55, fig. S7C and table S3). Since optimal debulking also appeared to be strongly associated with survival in a univariate analysis in this dataset (fig. S7D and table S3), we separated patients who were optimally debulked, defined as maximum residual tumor diameter < 1 cm, or sub optimally debulked. *BRCA* mutational status significantly correlated with better overall survival in the subgroup of patients who were optimally debulked (HR = 0.26, *P* = 0.019, Fig. 5B and table S3). In this patient subgroup, *BRCA1* methylation showed an intermediate effect that was not statistically significant (R = 0.45, Fig. 5B), whereas TDP status and HRD status did not associate with improved survival, regardless of optimal debulking (Fig. 5B).

The intermediate response observed in the UW *BRCA1*meth subset indicated that a subset of patients with *BRCA1*meth cancers did experience longer overall survival. We therefore examined if the degree of *BRCA1* promoter methylation could have a significant impact on therapeutic response, similar to what observed in the PDX TNBC cohort. We employed MS-ddPCR and reassessed the *BRCA1* methylation status of the cancer genomes in the UW OvCa cohort in a quantitative manner. We found that the degree of *BRCA1* methylation had a strong and significant negative correlation with *BRCA1* expression (r = −0.82, *P* = 0.0006, fig. S7E). Based on a methylation threshold of 70%, low-methylation cancers showed *BRCA1* expression akin to those found in non*BRCA1* cancers, wehereas cancers with high-methylation showed a reduction in *BRCA1* expression to less than 10% of what observed in non*BRCA1* cancers, (*P* = 3.1E-8, fig. S7F). Due to the limited number of cases in each methylation subgroup, no definitive conclusion could be made regarding overall survival in patients with OvCa relative to the amount of *BRCA1* promoter methylation, although the two patients with partial methylation had a particularly poor survival outcome (fig. S7G).

Adequacy of surgical cytoreduction is a known powerful prognostic factor in OvCa outcomes. Since the AOCS dataset did not have debulking information, we sought another large data set upon which we could validate the interaction of *BRCA* status and overall survival, while controlling for adequacy of surgical cytoreduction. The TCGA ovarian cancer cohort comprises 314 primary high grade serous ovarian carcinomas from patients that went on to receive carboplatin-based chemotherapy and with known *BRCA* status and, in the majority of cases, debulking status (25). Survival analysis of the full dataset confirmed a significant association between *BRCA* mutational status and better overall survival (HR = 0.053, *P* = 0.0003; fig. S7H), which was maintained after correcting for several outcome-associated clinical variables, including optimal debulking, race and age (table S3). On the other hand, *BRCA1*meth did not appear to provide any survival benefit (HR = 0.92, fig. S7H).

As expected, debulking status was the clinical variable most significantly associated with overall survival in the full dataset (*P* = 0.001, fig. S7I). When we restricted the survival analysis to the subset of patients in the TCGA OvCa cohort that received optimal debulking, we confirmed that patients with *BRCA*mut OvCas had better overall survival (HR = 0.54, *P* = 0.006, Fig. 5B and table S3), whereas *BRCA1* methylation status did not provide any benefit in terms of overall survival, even in this subgroup of patients with optimally debulked cancers. Thus, across all three OvCa cohorts examined and like in TNBC, *BRCA* deficiency by pathogenic mutations, but not *BRCA1* promoter methylation, was significantly associated with improved response to platinum-based therapy.

### In patients with non*BRCA* TNBC and OvCa, optimal response to the platinum/taxane chemotherapeutic combination is associated with an enhanced immune signature

**Fig. 6.**
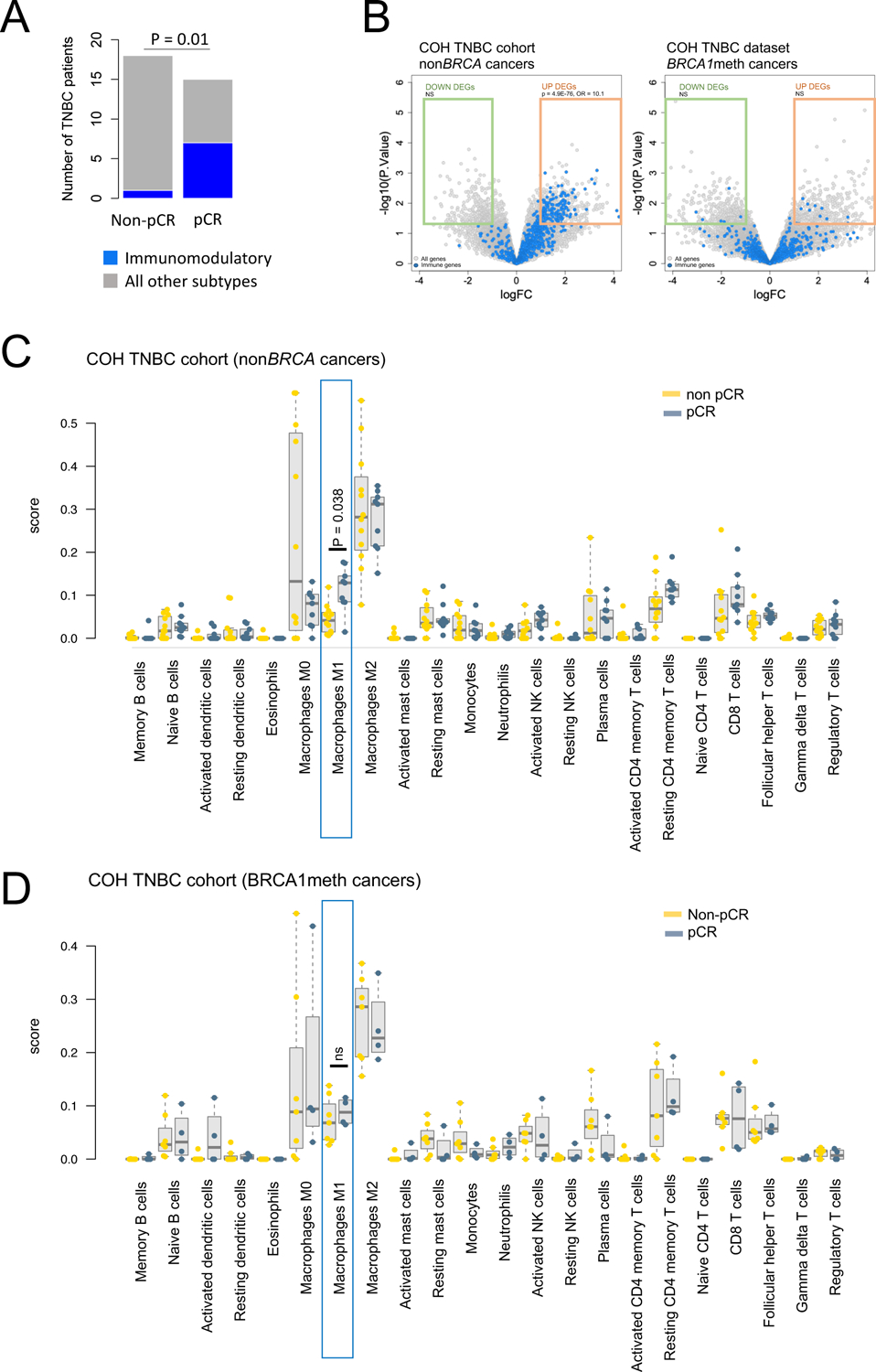
Immune expression signals and therapeutic response in non*BRCA* and *BRCA1*meth TNBCs. **(A)** TNBCtype classification of the COH TNBCs based on the six TNBCtype transcriptional subtypes, showing a significant enrichment of immunomodulatory subtype among patients who achieved pCR. Unclassified tumors were not included in the statistical analysis. *P* values were determined by Fisher’s exact test. **(B)** Volcano plots of differential gene expression between COH TNBC patient who achieved pCR and those who did not. Significantly differentially expressed genes (DEGs, *P* < 0.05 and absolute log2 fold change > 1), are boxed in green (down-regulated) and orange (up-regulated). Immune genes are shown in blue. Enrichment of immune genes within the down- and up-regulated differentially expressed genes was computed by Fisher’s exact test. **(C-D)** Comparison of cancer CIBERSORT scores between patients who achieved and did not achieve pCR (in blue and yellow, respectively), in the COH non*BRCA* TNBC (**C**) and *BRCA1*meth TNBC (**D**) cohorts. M1 macrophage scores are highlighted by blue boxes. *P* values were determined by Mann-Whitney test and Bonferroni correction for multiple testing.

We next sought to identify features associated with clinical response for patients with non*BRCA* cancers, starting with the COH TNBC cohort. Because we found no gene alterations, other than those affecting the *BRCA* genes, that were predictive for chemo-responsiveness in this cohort, we looked for transcriptional signals that associated with sensitivity to chemotherapy. The TNBC type classification described by Chen et al. segregates TNBCs into six transcriptional subsets, including a specific immunomodulatory subtype characteristic of TNBCs with a high degree of immune cell infiltrates (26). We found a significant enrichment for the immunomodulatory subtype in cancers from patients who achieved pCR (OR = 13.6, *P* = 0.01, Fig. 6A), but not for any of the other expression-based subtypes (table S3). Importantly, this enrichment was maintained when the analysis was restricted to the subset of PAM50 basal cancers (table S3). We then looked for specific expression profiles associated with better response across the entire TNBC cohort and found that the genes significantly over-expressed in TNBCs from patients who achieved pCR were highly enriched for immune response-related pathways, such as adaptive immunity and interferon signaling (fig. S8A), with a highly significant proportion of over-expressed genes annotated as immune genes (*P* = 6.0E-76, fig. S8B). When we sub-grouped TNBCs based on their *BRCA* status, the association between increased expression of immune genes and a higher pCR rate was only found in the non*BRCA* TNBC subset, but not in the *BRCA1*meth subset (Fig. 6B). On the other hand, since nearly all patients with *BRCA*mut cancers achieved pCR (8/9), elevated immune gene expression did not provide any added predictive value in this cancer subset. This suggests that the detected immune signal may be specifically linked to better response on a *BRCA*-proficient background.

We then asked if the observed immune signal could be parsed out into different types of infiltrating immune cells. To this end, we applied the CIBERSORT computational approach and assessed the relative abundance of 22 distinct immune cell types for each TNBC in the COH TNBC cohort with RNAseq expression profiling. The only significant association from the comparison of the CIBERSORT-generated scores between pCR and non-pCR subgroups was an increase in M1 macrophage scores (*P* = 0.038, Fig. 6C). Similar to the genetic analysis of differential gene expression described above, this association was not found when analyzing the subset of *BRCA1*meth TNBCs (Fig. 6D).

To assess if a similar trend was also observed in OvCa, we performed a multivariate analysis of all the optimally debulked OvCa cases in the TCGA OvCa and UW OvCa cohorts analyzed herein and assessed the contribution of the CIBERSORT M1 score as a continuous variable to overall survival while correcting for the original cancer cohort. In this aggregate dataset, we found a significant association between increasing M1 scores and longer overall survival only in patients with non*BRCA* OvCas (HR = 0.81, *P* = 0.047, fig. S8C), but not in those with either *BRCA*mut or *BRCA1*meth OvCas (fig. S8C). As the association of specific immune expression phenotypes with better survival in OvCa has been previously investigated with contrasting outcomes (25, 27–29), our analyses suggest that the beneficial effect of an augmented immune signature may be limited to non*BRCA* cancers, and potentially driven by M1 macrophage effects.

**Fig. 7.**
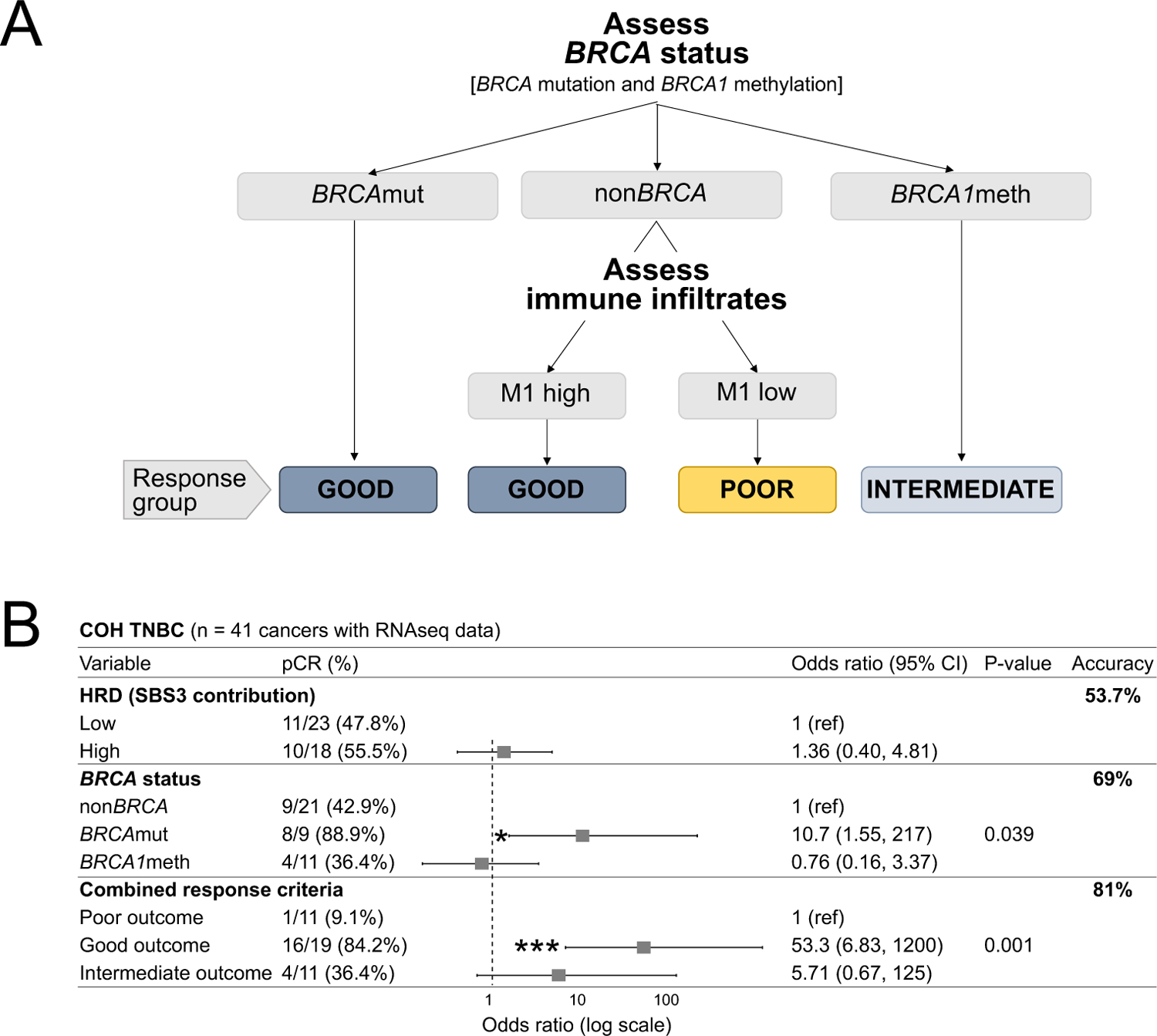
A decision tree for predicting response of patients with cancer to platinum-taxane combination chemotherapy. **(A)** Schematic of the proposed decision tree for optimal prediction of TNCB and OvCa patient response to platinum-taxane combination chemotherapy. M1 refers to the macrophage M1 signal computed using CIBERSORT, with high and low defined based on the median score value for the non*BRCA* subset of cancers**. (B)** Odd ratios of pCR for the COH TNBC cohort, with patients stratified based on either *BRCA* status alone, HRD-low or HRD-high, or the combined response criteria (computed as described in (**A**)) Odds ratio with 95% confidence intervals and *P* values were computed using logistic regression.

Taken together, our results strongly suggest that by considering *BRCA* status, as well as the expression of an immune signature in non*BRCA* cancers, we might be able to better predict chemo-responsiveness than by using *BRCA* mutational status alone or by assessing the HRD status in both TNBC and OvCa. To model this possibility, we developed a combined predictor that could better correlate with outcomes to platinum-based chemotherapy based on the presence of *BRCA* pathogenic mutations and, in non*BRCA* cancers, on the strength of the M1 macrophage transcriptional signal as indicated in the schematic in Fig. 7A. We compared the predictive performance of our combined response criteria with that of *BRCA* status alone and of HRD status. We applied this to the COH TNBC cohort which had the clearest metric for response, pCR. We found that 84.2% (16/19) of the patients predicted to have optimal therapeutic outcomes based on our combined response criteria did achieve pCR. On the other end, only 9.1% (1/11) of the patients predicted to be poor responders achieved pCR. When considered altogether, the accuracy of our algorithm in predicting pCR was 81% (Fig. 7B). By contrast, the predictive accuracy of *BRCA* status alone and of HRD status were 69% and 53.7%, respectively (Fig. 7B).

We then compared the three modes of patient stratification across the three OvCa cohorts examined in this study, using Cox proportional hazard ratio test statistics to examine overall survival trends. In each of the three cohorts, the response outcomes predicted by our proposed combined response criteria outperformed the predictive power of *BRCA* status alone and of HRD status, as indicated by increased statistical significance and generally lower hazard ratios (fig. S8D).

These findings further support our notion that BRCA deficiency status computed using genome-based measures of HRD is not a good predictor of chemotherapeutic response, and that additional patient stratification focused on *BRCA* mutation and methylation status and immune transcriptional profiles, the M1 macrophage signature in particular, could provide improved clinical prediction in TNBC and OvCa if prospectively validated.

## DISCUSSION

A major focus of this study was to explore the causes for the inconsistencies in the clinical predictive value of *BRCA* status and HRD scores in defining chemotherapeutic response in TNBC and OvCa. These inconsistencies were puzzling given the existing knowledge of BRCA1, BRCA2, their functions in homologous recombination, and the association of BRCA disruption with sensitivity to DNA damaging agents such as platinum compounds (19, 20, 30, 31). Particularly controversial is the predictive value of *BRCA1* promoter methylation. A comprehensive meta-analysis of 34 studies encompassing 7,986 treated patients with OvCa showed that *BRCA*mut cancers were associated with better survival compared to nonBRCA cancers, but that *BRCA1*meth cancers had no such association (16). Similarly, in the two-arm randomized TNT clinical trial, Tutt et al. reported that, contrary to the presence of *BRCA1* germline mutations, neither *BRCA1* methylation nor Myriad HRD assessment were associated with better response to carboplatin compared to docetaxel in metastatic TNBC (12). The contrasting outcomes of tumors with these markers contradict the biochemical equivalence of the *BRCA1*meth and *BRCA1*mut statuses. Our investingations consistently show across all the clinical TNBC and OvCa cohorts examined that *BRCA1*meth cancers indeed have a lower objective response to platinum compounds than *BRCA1*mut cancers despite the association of *BRCA1* promoter methylation with a reduction in *BRCA1* RNA and with patterns of TDP type 1 formation identical to those found in *BRCA1*mut cancers.

Our work resolves this conundrum by showing that *BRCA1* methylation is a functionally plastic state within the cancer cell and that it can be rapidly lost upon chemotherapy exposure. This results in the restoration of *BRCA1* expression and downstream resistance to platinum compounds. The working model of this process is that cytotoxic chemotherapy induces the specific demethylation of one *BRCA1* promoter allele in a stochastic fashion in subpopulations of cells within a tumor. Each subsequent cycle of chemotherapy not only demethylates more cells at the *BRCA1* promoter, but also progressively enriches for these partially methylated cells as the more chemosensitive fully *BRCA1* methylated cancer cells are progressively eliminated. This process of epigenetic recruitment and clonal selection occurs very rapidly in PDX tumors and explains many of the clinical observations. The use of PDX models of *BRCA1*meth TNBC was experimentally important in that, to our knowledge, there is no established cancer cell line with methylation of both *BRCA1* promoter alleles.

Our study also can explain why diagnostics such as measures of HRD and immune biomarkers are inconsistent in predicting outcome in TNBC and OvCa clinical trials. The utility of HRD metrics appears to be dependent on the ratio of *BRCA*mut to *BRCA1*meth cancers in any cohort: only studies with a high ratio would show a predictive value associated with HRD status, since *BRCA1*meth cancers with characteristically high HRD scores show no preferential response to platinum agents. Consistent with this, the sole cohort we examined that exhibited an association of HRD with response was the TCGA OvCa cohort which had the highest ratio of *BRCA*mut to *BRCA1*meth cancers of all the cohorts studied.

Similarly, we found that the salutary effects of a high immune score rested primarily in the subgroup with non*BRCA* cancers, and is not a general feature of all TNBCs and OvCas. Here, again, the ratio of non*BRCA* cancers to *BRCA1* deficient cancers will likely determine how effective immune scores are in predicting chemotherapeutic response. Most studies examining the question of immune signatures in TNBC and OvCa did not stratify patients based on these outlined BRCA states (28, 29, 32). We have further refined the key immunological configuration for a good response to be an elevation of the M1 macrophage expression cassette, which reflects activated tumor associated macrophages that exert an anti-tumor cytotoxic effect (33). Moreover, recent evidence showed that M1 macrophage polarization in the tumor microenvironment is associated with the best prognostic outcome for patients with OvCa (34). It is not clear why this immunological effect on therapeutic outcome is particularly impactful in non*BRCA* cancers since this immune signature appears also in BRCA1 deficient cancers. One possibility is that the benefits of a *BRCA*mut status in chemosensitivity outweighs any benefit accrued through an immunological mechanism. Regardless of the mechanism, using this stratification of *BRCA* status and immune configuration, we constructed a putative predictive model for response to platinum in TNBC and OvCa that outperforms the use of HRD metrics, or of simply ascertaining *BRCA*mut status.

Our study has several limitations. The sample sizes of the patient cohorts with TNBC and OvCa we sequenced were small, albeit selected to address the specific question of *BRCA* status and platinum sensitivity. The remainder of the clinical analyses were performed retrospectively on heterogeneous cohorts treated with a variety of chemotherapeutic agents. Moreover, the detailed genomic longitudinal data we provided were solely in PDX models which are imperfect replicas of the human condition. Therefore, the most rigorous validation of our findings would be a large prospective clinical trial structured to answer the differential impact of neoadjuvant chemotherapy between patients with *BRCA*mut and *BRCA1*meth cancers and to test the conversion of *BRCA1*meth cancers to the partially methylated state over the course of therapeutic cycles. Given that the combination of checkpoint inhibitors and chemotherapy has become the recommended neoadjuvant regimen in TNBC despite an increase in adverse drug events, the ability to deescalate a toxic combination in a subset of patients with TNBC and OvCa potentially identified by our decision tree would have a meaningful impact on cancer survivors.

## MATERIALS AND METHODS

### Study Design.

In this study we utilized in-house generated and publicly available genomic datasets, PDX models and genetically engineered cell lines to address the value of HRD status, *BRCA* status and immune transcriptional signatures as potential biomarkers of platinum-response. The sequencing data generated in this study include three WGS datasets and two RNAseq datasets, all of which were deposited in the European Genome-Phenome Archive (EGA). For the UW OvCa cohort, a total of 63 cancer samples were selected to represent diverse outcomes of patient survival as well as the three different *BRCA1/2* states examined in this study, but independently of any other genetic or clinical features. For each of the other cohorts examined, we analyzed all the cancer samples that were available to us. No statistical methods were used to predetermine the sample size. No exclusion criteria were preestablished, and no data were excluded from analyses. All investigations involving human specimens were performed after approval by the Institutional Review Board at each institution, and all subjects provided voluntary written informed consent. In vivo PDX studies were conducted to compare the platinum-response rates across different genomic and epigenomic states and to assess changes in *BRCA1* promoter methylation following platinum therapy. Animals were randomized into treatment and control groups, with a minimum of three replicate animals per group. No statistical methods were used to predetermine the sample size. All animal protocols were reviewed and approved by the appropriate institutional Animal Care and Use Committee (IACUC) before study initiation. To deconvolute the *BRCA1*mut-specific response to cisplatin versus docetaxel, we performed in vitro IC_50_ experiments on two isogenic cell lines that differ for their BRCA1 deficiency status. We carried out four biological replicates of each experiment, each one comprising three technical replicates. Changes in *BRCA1* gene expression were assessed by qPCR. Experiments were conducted in technical triplicates, and we always included one or more DNA samples from non*BRCA* cancers to be used as calibrators. *BRCA1* promoter methylation was assessed by either MSP, for a qualitative assessment of the methylation status, or MS-ddPCR, for a precise quantification of the degree of methylation. In both instances, human methylated and non-methylated DNA standards were included as controls for effective bisulfite conversion and PCR amplification.

### Tumor cohorts and WGS

Three new human tumor WGS datasets were generated as part of this study, as listed in table S1. All investigations involving human specimens were performed after approval by the Institutional Review Board at each institution, and all subjects provided voluntary written informed consent. *City of Hope National Medical Center (COH TNBC) cohort*. This cohort comprises 42 female patients with pathologically confirmed diagnosis of locally advanced and inflammatory TNBC, who were enrolled in a phase II trial testing the safety and efficacy of carboplatin and NAB-paclitaxel in the neoadjuvant setting (NCT01525966, (17)) at the City of Hope Comprehensive Cancer Center. Specimens from pre-treatment tumor biopsies were snap-frozen in RNA-later solution. DNA and RNA were isolated from the same tissue fragment using the QIAGEN AllPrep DNA/RNA Mini Kit. *PDX cohort*. This dataset comprises 36 PDX TNBC models established in the Advanced In Vivo Models Core at the Baylor College of Medicine (BCM), under the direction of Prof. M. T. Lewis (35), and with known response to single agent carboplatin and docetaxel, assessed as part of a PDX preclinical study (https://pdxportal.research.bcm.edu/). Briefly, for each model, appropriately sized female mice cohorts were established through orthotopic tumor transplantation and when tumors reached a volume of ~150–200 mm^3^, mice were randomly assigned to one of three study arms (control, carboplatin or docetaxel) and subjected to a four-week regimen of weekly intraperitoneal doses of either vehicle, carboplatin at 50 milligrams per kilograms of body weight (mg/kg) or docetaxel at 20 mg/kg. Tumor growth was monitored by biweekly tumor volume measurements using calipers, and datapoint collected up to seven days following the administration of the last dose of each agent (day 28) were used for the response analysis. For the WGS analysis, DNA was isolated from snap-frozen tumor tissue fragments using a QIAGEN AllPrep DNA/RNA Mini Kit. The PDX TNBC cohort also includes seven TNBC PDX models from the Jackson Laboratory (JAX) PDX Resource, established under protocol 12027 approved by The Jackson Laboratory Institutional Animal Care and Use Committee (IACUC) before study initiation. Details regarding the establishment and therapeutic characterization of these models have been previously published (36). Briefly, tumors were implanted subcutaneously into the flank of NOD-*scid*-IL2 receptor gamma null female mice (NSG). Individual tumor-bearing mice were then randomized into one of three study arms (control, cisplatin or docetaxel) of at least eight animals each on an accrual basis when tumors reached a volume of 150 mm^3^ and subjected to a three-week regimen of weekly intraperitoneal injections of either vehicle, cisplatin at 2 mg/kg or docetaxel at 20 mg/kg. Biweekly tumor volume measurements up to seven days following the administration of the last dose (day 21) were analyzed as described below. Additional information and data for these PDX models are publicly available from the PDX Portal hosted by Mouse Tumor Biology Database (MTB; http://tumor.informatics.jax.org/mtbwi/pdxSearch.do). *University of Washington (UW OvCa) cohort*. A total of 63 serous ovarian carcinomas were selected from the Gynecologic Oncology Tissue Bank established by Prof. E. M. Swisher at the University of Washington, Seattle, WA, to represent diverse outcomes of patient survival as well as the three different *BRCA1/2* states examined in this study, but independently of any other genetic or clinical features. Patients were treated with a combination of carboplatin and paclitaxel. DNA and RNA were isolated as previously described (14).

WGS libraries were generated using either a KAPA Hyper Prep Kit (COH TNBC cohort) or a TruSeq DNA PCR -Free kit (UW OvCa and PDX cohorts), according to manufacturer guidelines and 150 bp paired-end sequence reads were generated using either the Illumina HiSeq X Ten system (COH TNBC cohort) or a NovaSeq 6000 system (UW OvCa and PDX cohorts). Raw sequencing data were aligned to the human GRCh37 reference genome using the Burrows-Wheeler Aligner (BWA) (37). In the case of the PDX cohort, potential mouse contaminant reads were removed by aligning the data to a combined reference genome of mouse (GRCm38/mm10) and human (GRCh37). Structural variant calls were generated using three different tools (Crest (38), Delly (39), and BreakDancer (40)), and high confidence events were selected when called by at least two tools and by requiring split-read support. In the absence of matched normal DNA samples to be used as controls, germline variants were identified as those that appear in the Database of Genomic Variants (DGV, http://dgv.tcag.ca/) or the 1,000 Genomes Project database (http://www.internationalgenome.org), as well as by filtering rearrangements identified across an internal panel of normal genomes. Single Nucleotide Variants were called by muTect (41), Strelka (42) and LoFreq (43). We considered high confidence mutations those identified by all three tools. In the absence of matched normal DNA, we used a contemporary normal DNA sample from the HapMap project (NA12878) that was prepped and sequenced using the same protocol as the cancer sample. Somatic mutations were considered those unique to the cancer genome. In addition, we removed mutations with MAF ≥ 1% as reported in the 1000 Genomes Project release 3 (1000 Genomes Project Consortium, 2012) or the Exome Aggregation Consortium (ExAC) server. Single base substitution signatures (SBS signatures) were extracted with the *MutationalPatterns* R package, using the COSMIC signatures v2 as the mutational-process matrix.

### TDP and HRD metrics.

TDP status for all WGS-cohorts analyzed in this study was ascertained as previously described (4). In the absence of matched germline genomic data for the in-house sequenced datasets (COH TNBC, PDX TNBC and UW OvCa cohorts), we were not able to measure HRD using published algorithms such as the copy-number-based HRD score proposed by Telli et al. (11) or the WGS-based HRDetect method described by Davies et al. (2). Instead, an HRD score was computed as the relative contribution of the SBS3 mutational signature to the genomic mutational burden and a threshold contribution 5% was used to separate HRD-low vs. HRD-high cancer genomes. For consistency, a similar approach was applied to identify HRD-low vs. HRD-high cancer genomes in the AOCS cohort, but using a higher threshold contribution of 20%. For the TCGA OvCa dataset, the HRD index was computed as the unweighted sum of measures of telomeric allelic imbalance (NtAI score), large scale transition (LST score), and loss of heterozygosity HRD-LOH score), as reported by Marquard et al. (44). Genomes with HRD scores > 42 were classified as HRD-high, as originally described in Telli et al. (11).

### RNAseq.

RNA-seq libraries for the COH TNBC cohort were generated using the KAPA Stranded mRNA-Seq kit (Roche) according to manufacturer’s instructions and were sequenced on a HiSeq4000 Illumina platform to generate 75 bp paired end reads. RNA-seq libraries for the UW OvCa cohort were generated using the KAPA mRNA Hyperprep kit (Roche) according to manufacturer’s instructions and were sequenced on a NovaSeq Illumina platform to generate 100 bp paired end reads. For both datasets, high quality unique paired-end reads were aligned to the GRCh38-based UCSC gene reference transcriptome using Bowtie2, and RSEM (45) was used to estimate the abundance of each individual gene. Upper quartile normalization was performed within each tumor sample after discarding genes with no counts. Finally, gene expression was adjusted using a percentile rank transformation. Analysis of differential gene expression was performed using the *limma* package in R. For the COH TNBC dataset, TNBC transcriptional subtypes were determined using the TNBCtype-4 tool as described in (46) and the *genfu* library in R for PAM50-based classification.

### Published tumor datasets.

#### AOCS cohort.

This cohort comprises 80 primary serous ovarian carcinomas that are part of the Australian Ovarian Cancer Study (AOCS). Their *BRCA1/2* status had been previously published (24). Structural variant and mutation calls were downloaded from the COSMIC data portal (data freeze version v78). Clinical data, RNAseq-based gene expression and methylation array (Illumina human methylation450K array) data were downloaded from the ICGC data Portal (https://dcc.icgc.org/) in August 2020.

#### TCGA OvCa cohort.

This dataset comprises 314 primary serous ovarian carcinomas from the TCGA ovarian cohort (25). Their BRCA*1/2* mutational status was obtained from cBioPortal (June 2020 download, (47, 48) and from previous publications (25, 49). *BRCA1* promoter methylation was computed based on Illumina human methylation27K array Beta-values downloaded from the UCSC Xena Browser in June 2020: the average Beta-value relative to the four probes mapping to the minimal promoter of the *BRCA1* gene (cg19531713, cg19088651, cg08993267, cg04658354) was computed and a threshold of 0.4 was selected to identify methylated cancers. Clinical data and microarray-based gene expression data were downloaded from the UCSC Xena Browser in June 2020.

### PDX response metrics.

Response to treatment across the entire PDX cohort was evaluated using two independent metrics that resulted in highly comparable outcomes, as detailed in tables S2 and S4. In the first instance, we took full advantage of the PDX model system and compared tumor growth rates between animals in the treatment and control arms. Briefly, the rate of tumor growth for each animal was computed by fitting a linear curve to the log-transformed tumor volumes, as previously described (50). This value was then compared to the average tumor growth rate of the animals in the control arm for each TNBC PDX model, to calculate the percentage difference in tumor growth rate relative to each individual animal. Finally, values corresponding to replicate animals were averaged to compare percentage reductions in tumor growth rates across the different genetic backgrounds. Binary response was determined by setting a threshold corresponding to 5% reduction in tumor growth rate in the treatment arm compared to the control arm. This percentage reduction corresponds to a 50% decrease in tumor volume for the animals in the treatment arm when compared to those in the control arm over a 14-day timeframe, assuming comparable tumor volumes between the two arms at the beginning of the study. Response was also assessed based on the percentage tumor volume change (ΔVol) at the end of treatment (day 28 for a 4-week regimen, day 21 for a 3-week regimen) compared with its baseline (day 0). The criteria for response were adapted from Response Evaluation Criteria in Solid Tumors (RECIST) criteria (51) and defined as follows: complete response, Δvol < −80%; partial response, −80% < Δvol < −30%; stable disease, −30% < Δvol < 20%; progressive disease, Δvol > 20%. The mode outcome for each group of replicate animals was reported as the response call for each PDX model examined, with models which demonstrated RECIST values equivalent to complete or partial response being designated as responders.

The TM00097 and TM01079 TNBC PDX models from the JAX PDX Resource were additionally analyzed to assess the effect of in vivo cisplatin treatment on *BRCA1* expression and promoter methylation. For each model, appropriately sized mice cohorts were established through subcutaneous tumor injection and when tumors reached a volume of ~150–300 mm^3^, mice were randomly assigned to one of the two study arms (control or cisplatin) and subjected to four weekly doses of either vehicle or cisplatin at 2 mg/kg. The TM00097 PDX model was also assessed for its response to the AC→T regimen, which consisted of three weekly doses of both doxorubicin (2 mg/kg) and cyclophosphamide (40 mg/kg), followed by three weekly doses of docetaxel (6 mg/kg). Following treatment, host mice were given a drug holiday until their tumors reached ~2000 mm^3^ in volume or, for tumors that shrunk following treatment, until they grew back to at least twice the size of their original pre-treatment volume, at which point they were harvested and assessed for *BRCA1* expression using qPCR and promoter methylation using MSP. All animal procedures employed for this additional study were approved by The Jackson Laboratory Institutional Animal Care and Use Committee (IACUC) under protocol number 12027.

### PDX-derived primary cultures.

Primary cell cultures were established from cryopreserved PDX tumor fragments as previously described (52). Briefly, tumor fragments were dissociated through incubation with collagenase for one hour, washed and plated on a layer of 3T3-J2 irradiated feeder cells and grown in the culture medium described by Liu et al. (53). Following in vitro expansion, single cells were isolated using flow cytometry and further expanded to establish individual clonal lines.

### Cell Culture and IC_50_ Determination.

The SUM149 and SUM149.B1.S* cell lines were a kind gift by Prof. C. Lord. SUM149 carries a pathogenic variant of the *BRCA1* gene (c.2288delT, p.N723fsX13) and has lost the wild type form of the gene. Its CRISPR/Cas9-derived daughter clone SUM149.B1.S* harbors an 80-bp deletion downstream of the parental mutation (c.[2288delT;2293del80]), which restores the open reading frame in the parental BRCA1 c.2288delT allele and encodes a functional 1836-amino acid-long BRCA1 protein (22). Both cell lines were authenticated by amplification and Sanger sequencing of the *BRCA1* locus harboring the original and secondary mutations and regularly tested for *Mycoplasma* contamination using the MycoAlert PLUS Mycoplasma Detection Kit (Lonza). The two cell lines were maintained in Ham’s F-12 Nutrient Mixture medium with 5% (vol/vol) FBS, 1% (vol/vol) Penicillin-Streptomycin, 0.01 mg/mL bovine insulin and 1 μg/ml hydrocortisone. For IC_50_ value determinations, cells were plated in 96-well plates at a density of 2 × 10^3^ cells per well. After 24 hours in culture, cisplatin or docetaxel (both from Selleck Chemicals) were added in triplicate wells to the culture medium in two-fold serial dilutions in the range of 0.05 to 205 μM (cisplatin), or 0.05 to 205 nM (docetaxel). Cells were incubated for 72 hours before assessing cell viability using a WST-8 assay (Dojindo Molecular Technologies, Inc.). Absorbance values were normalized to control wells (medium only), and IC_50_ values were calculated using the IC_50_ R package (54). Four independent biological replicate experiments were carried out for each cell line and each treatment, and the Student’s t-test statistic was used to determine the significance of the difference between the average IC_50_ values relative to the two cell lines.

### *BRCA1* promoter methylation analysis.

*BRCA1* promoter methylation status for the COH TNBC and PDX datasets was determined by methylation-specific PCR (MSP). Briefly, 100 ng of genomic DNA was subjected to bisulfite conversion using the EpiTect bisulfite kit (QIAGEN). Following clean-up, the converted DNA was used as template for two separate PCR reactions to amplify the proximal *BRCA1* gene promoter region with two sets of primers specific for either unmethylated (forward: 5’-TTG GTT TTT GTG GTA ATG GAA AAG TGT-3’; reverse: 5’-CAA AAA ATC TCA ACA AAC TCA CAC CA-3’) or methylated DNA (forward: 5’-TCG TGG TAA CGG AAA AGC GC-3’; reverse: 5’-AAA TCT CAA CGA ACT CAC GCC G-3’). PCR products were then loaded on a 2% agarose gel and analyzed to visualize the amplification products. Samples with visible amplification of the methylation-specific PCR template were considered methylated. Either DNA from the *BRCA1*-methylated HCC38 TNBC cell line or CpGenome^™^ Human Methylated and Non-Methylated DNA Standards (Sigma-Aldrich) were included in each bisulfite conversion reaction as controls. *BRCA1* promoter methylation for the UW OvCa dataset was determined by bisulfite conversion using the Zymo Research EZ DNA Methylation-Direct kit followed by MSP, as previously described (14). Methylation specific droplet digital PCR (MS-ddPCR) was carried out to quantify the levels of *BRCA1* promoter methylation in the subset of methylated cancers from the COH TNBC, PDX TNBC and UW OvCa cohorts, as previously described (21). *BRCA1* methylation values relative to the UW OvCa cohort were corrected for neoplastic cellularity as assessed based on H&E staining. For the UW OvCa cohort a methylation threshold of 70% was applied to identify complete vs. partial methylation, as previously published (21). In the absence of H&E assessment, potential normal tissue contamination for the COH TNBCs was accounted for by lowering the threshold for complete methylation to 60%, This threshold modification was based on the median amount of non-cancer tissue assessed in OvCa samples that underwent neoplastic cellularity assessment, as noted above (~10%). Abrogation of BRCA2 through epigenetic silencing is an extremely rare event (55, 56) and we did not find any instances of *BRCA2* promoter methylation in our analysis of the AOCS and TCGA cohorts. Therefore, we did not incorporate *BRCA2* promoter methylation analysis in this study.

### qPCR analysis of *BRCA1* gene expression.

Up to 500 ng of RNA was reverse transcribed using Maxima reverse transcriptase mix (Fermentas). Each qPCR reaction was performed using 15 ng of cDNA as template, 0.5μM forward and reverse primers and SYBR-Green PCR Mix (Fermentas), according to the manufacturer’s instruction and using the ABI7500 system (ABI). Expression of the *BRCA1* gene was evaluated using the following primers: forward: 5’-TTG CAG TGT GGG AGA TCA AG-3’; reverse: 5’-CGC TTC TCA GTG GTG TTC AA-3’. The *SRP14* gene was used as the housekeeping control (forward: 5’- AGG GTA CTG TGG AGG GCT TT-3’; reverse: 5’- GCT GCT GCT TTG GTC TTC TT-3’). Every qPCR assessment was performed in three replicate wells and the average of the Ct values relative to the three technical replicates was used to compute the relative *BRCA1* expression using the delta-delta Ct method.

### PCR validation of the *STAT3/NBR2/BRCA1* fusion gene and fusion transcript junctions.

To confirm the tandem duplication breakpoint relative to the *STAT3/NBR2* gene fusion observed in the WHIM75 sample, primers were designed to amplify a ~400 base pair genomic region surrounding the estimated breakpoint junction (forward: 5’-TCT CCT CTC TGG TCC CTT GA-3’; reverse: 5’-TTT TGG TTT CCA ACC AGA GC-3’). PCR products were visualized on a 2% agarose gel and, following PCR purification, sequenced using Sanger sequencing, to identify the exact sequence of the breakpoint junction. To confirm the expression of the *STAT3/BRCA1* fusion transcript, 500 ng of RNA was reverse transcribed using Maxima reverse transcriptase mix (Fermentas) and amplified with PCR primers surrounding the estimated fusion junction (forward: 5’-AGC AGC ACC TTC AGG ATG TC-3’; reverse: 5’-GAA GGC CCT TTC TTC TGG TT-3’). PCR products were visualized and sequenced as described above.

### Analysis of immune cell infiltrates.

CIBERSORT is a deconvolution algorithm that allows to characterize the immune infiltrate cell composition of complex tissues, including tumor tissues, from their bulk gene expression profiles (57). We applied the CIBERSORT relative analytical tool from the Alizadeh lab (https://cibersort.stanford.edu) and the default LM22 signature matrix to quantify 22 different human hematopoietic cell types across our four expression datasets. For RNAseq-based gene expression datasets, we measured gene abundance in Transcripts Per Kilobase Million and disabled quantile normalization, as recommended. We then compared CIBERSORT-generated abundance scores across responders and non-responders using a logistic regression model.

### Statistics.

Unless otherwise stated, statistical analyses were conducted, and graphics produced using the R statistical programming language version 3.6.1 (www.cran.r-project.org), using built-in and freely available R packages. Logistic regression analyses to assess the association between therapy response as a binary outcome and clinical/genetic variables in the COH TNBC cohort were performed using the *glm* function in R, specifying a binomial distribution and utilizing *logit* as the link function. Response associations in the PDX TNBC cohort were evaluated using the Fisher’s Exact test. In survival analyses (AOCS, UW OvCa and TCGA OvCa cohorts), the *coxph function* (from the *survival* R package) was applied to compute the Cox proportional hazards regression model, using the *Surv* function to create the survival object. All other statistical tests employed are specified in the appropriate Result sections and corresponding figure legends.

## Supplementary Material

Supplementary_Figures1-8

Supplementary_Tables_1-5

## Data Availability

All data associated with this study are present in the paper or supplementary materials. Raw sequencing fastq files relative to the WGS and RNAseq datasets generated as part of this study have been deposited to the European Genome-Phenome Archive and are available under controlled access (COH TNBC WGS: EGAD00001008617; COH TNBC RNAseq: EGAD00001008542; PDX TNBC WGS: EGAD00001008535; UW OvCa WGS: EGAD00001008581; UW OvCa RNAseq: EGAD00001008542). All of the PDX models analyzed in this study are available from either the Baylor College of Medicine PDX portal (BCM PDX models, n = 36, https://pdxportal.research.bcm.edu/), or the the PDX Portal hosted by the Mouse Tumor Biology Database (JAX PDX models, n = 7, http://tumor.informatics.jax.org/mtbwi/pdxSearch.do).
