# Supplementary material for: Genomic and epigenomic BRCA alterations predict adaptive resistance and response to platinum-based therapy in patients with triple negative breast and ovarian carcinomas
Source: Sci Transl Med. Author manuscript; Available in PMC 2022 Oct 21. (PMC9585706; doi:10.1126/scitranslmed.abn1926)
Supplement: Supplementary_Figures1-8 [file NIHMS1841907-supplement-Supplementary_Figures1-8.docx]

**Supplementary Materials**

**Fig. S1. Genomic features as a function of *BRCA1* status in the COH TNBC cohort. (A)** Number of tandem duplications across *BRCA1*meth, *BRCA1*mut and non*BRCA1* cancer genomes; the *P* value was determined by Student’s t-test (two-tailed). Box plot elements: center line, median; box limits, lower and upper quartiles; whiskers extend up to one and a half times the interquartile range; ns, not significant. **(B)** Tandem duplication span size distributions across *BRCA1*meth, *BRCA1*mut and non*BRCA1* TNBC genomes; the *P* value was determined by Mann-Whitney test.

**Fig. S2. Platinum response as a function of genomic configurations in the PDX TNBC cohort. (A-B)** Percentage of platinum responders as a function of TDP status (**A**) or HRD status (**B**) in the PDX cohort; *P* values was determined by Fisher’s exact test.

**Fig. S3. Patterns of complete and partial *BRCA1* methylation in TNBC PDX tissues. (A)** MSP results for three exemplary TNBC PDX models, showing three different *BRCA1* methylation patterns at the proximal promoter: complete and partial methylation, and absence of methylation. U, unmethylated PCR product; M, methylated PCR product. (**B**) Scatter plot of the degree of *BRCA1* methylation and the percentage of growth rate reduction relative to the 16 *BRCA1*meth PDX models in the PDX TNBC cohort. A smooth local regression line with 95% confidence interval was drawn using the *geom_smooth* function in R (method = loess). The Pearson correlation coefficient (r) and its corresponding *P* value are shown.

**Fig. S4. *G*enomic features of acquired resistance to platinum-based therapy in two cases of *BRCA1*meth cancers. (A)** Overview of the genomic region on *Chr17* hosting the *STAT3, NBR2* and *BRCA1* genes (top), of the de novo tandem duplication in the WHIM75 cancer genome (middle), and of the resulting gene fusion between *STAT3* and *NBR2* (bottom). Individual exons are represented by vertical lines and the tandem duplication breakpoint is indicated by a dashed line. Genomic coordinates are based on the GRCh38 genome built. **(B)** Gel image of the genomic DNA PCR using primers spanning the *STAT3/NBR2* gene fusion junction. Only WHIM75 shows an amplification signal. **(C)** Sequence analysis of the *STAT3/NBR2* tandem duplication breakpoint junction in the WHIM75 PDX. Sequence alignments relative to the *NBR2* and *STAT3* intronic regions involved in the fusion are shown in blue and green, respectively. The novel gene fusion sequence is shown in the middle row in bold highlight. Grey highlights represent the region of microhomology at the breakpoint junction. **(D)** Gel image of the reverse transcription PCR using primers spanning the *STAT3/BRCA1* fusion transcript junction. Only WHIM75 shows an amplification signal. **(E)** Summary of structural variations and tandem duplication span sizes relative to the paired primary and recurrent OvCa genomes from donor AOCS-091 (AOCS cohort).

**Fig. S5. Analysis of *BRCA1* expression levels in control vs. cisplatin-treated *BRCA1*meth PDXs and PDX-derived clonal cell lines. (A)** *BRCA1* gene expression (qPCR) for vehicle tumors and cisplatin- or docetaxel-treated recurrences relative to four TNBC PDX models with different *BRCA* backgrounds. All the data is normalized to the average *BRCA1* expression of control tumors from the non*BRCA* PDX #TM00096. *P* values were determined by Student’s t-test (two-tailed). **(B)** qPCR of *BRCA1* gene expression across the fifteen single cell clonal expansions established from tumor residuals/relapses of the TNBC PDX model #TM01079. *BRCA1* expression is normalized to the *BRCA1* expression of the cisplatin-arm-derived primary cell cline, which is represented in dark blue (CIS pool). Data are presented as mean values and standard errors of three technical replicates. The *P* value was determined by Student’s t-test (two-tailed).

**Fig. S6. Assessment of *BRCA1* methylation and expression in a *BRCA1*meth TNBC PDX treated with AC🡪T *in vivo*. (A)** Tumor growth curves relative to the TNBC PDX model TM00097. Black and grey arrow heads indicate the timing of the three weekly doses of the doxorubicin/cyclophosphamide combination and of docetaxel, respectively. **(B)** MSP results for vehicle tumors and AC🡪T-treated tumor recurrences relative to PDX #TM00097. **(C)** qPCR of *BRCA1* gene expression across vehicle tumors and AC🡪T-treated tumor recurrences*. BRCA1* expression is normalized to the average *BRCA1* expression of control tumors from the non*BRCA* PDX #TM00096. The *P* value was determined by Student’s t-test (two-tailed).

**Fig. S7. Analysis of therapeutic response in the OvCa cohorts. (A)** AOCS cohort: percentage of tumors with BRCA1 abrogation relative to TDP status. P by logistic regression. **(B)** UW OvCa cohort: percentage of tumors with BRCA1 abrogation relative to TDP status. The *P* value was determined by logistic regression. (**C-D**) UW OvCa cohort. Overall survival of patients stratified based on *BRCA* status (**C**) or debulking status (**D**). (**E**) Scatter plot of *BRCA1* gene expression (RNAseq log 2 values) and *BRCA1* promoter methylation (based on MS-ddPCR estimates) for the subset of 13 *BRCA1* methylated UW OvCas with available expression data. A smooth local regression line with 95% confidence interval was drawn using the *geom_smooth* function in R (method = loess). The Pearson correlation coefficient (r) and its corresponding *P* value are shown. (**F**) *BRCA1* gene expression (RNAseq) across the UW OvCa cohort as a function of *BRCA1* status. The *P* value was determined by Student’s t-test (two-tailed). (**G**) UW OvCa cohort. Overall survival of patients stratified based on *BRCA* status, including the separation between *BRCA1*meth high and low. **(H-I)** TCGA OvCa cohort. Overall survival of patients stratified based on *BRCA* status (**H**) or debulking status (**I**). For all the survival analyses, the Cox proportional hazards regression model was used to compute hazard ratios (HR) with 95% confidence intervals (in brackets), and their corresponding *P* values.

**Fig. S8. Associations between immune transcriptional profiles, *BRCA* status and patient response. (A)** COH TNBC cohort. TOP 20 Gene Ontology biological process terms significantly enriched in the set of up-regulated genes in TNBCs from patients who achieved pCR vs. those who did not. Terms are sorted by increasing *P* value and their relative fold enrichment is indicated on the x axis. The number of significant genes for each category is depicted within each bar. **(B)** COH TNBC cohort. Volcano plot of differential gene expression between patients who achieved pCR vs. those who did not from the entire COH TNBC cohort. Significantly differentially expressed genes (DEGs, p-value <0.05 and absolute log2 fold change > 1), are indicated by green (down-regulated) and orange (up-regulated) boxes. Immune genes are shown in blue. Enrichment of immune genes within the up-regulated differentially expressed genes was computed by Fisher’s exact test. **(C)** Multivariate COX proportional hazard model for overall survival of patients with optimally debulked OvCa (UW OvCa and TCGA OvCa cohorts), stratified based on *BRCA* status. Hazard ratios were computed using CIBERSORT M1 scores (as a continuous variable) and cancer cohort of origin as variables. Significant *P* values are highlighted in red. (**D**) Hazard ratios for overall survival of patients with OvCa in the UW OvCa, AOCS and TCGA OvCa cohorts, stratified based on either HRD-low vs. HRD-high, *BRCA* status alone, or the combined response criteria (computed as described in **Fig. 7A**). Hazard ratio with 95% confidence intervals and *P* values were computed using the COX proportional hazard test. * P < 0.05; ** P < 0.01; *** P < 0.001.
